# Optimal Signal Processing in Small Stochastic Biochemical Networks

**DOI:** 10.1371/journal.pone.0001077

**Published:** 2007-10-24

**Authors:** Etay Ziv, Ilya Nemenman, Chris H. Wiggins

**Affiliations:** 1 College of Physicians and Surgeons, Columbia University, New York, New York, United States of America; 2 Department of Biomedical Engineering, Columbia University, New York, New York, United States of America; 3 Department of Applied Physics and Applied Mathematics, Columbia University, New York, New York, United States of America; 4 Center for Computational Biology and Bioinformatics, Columbia University, New York, New York, United States of America; 5 Computer, Computational and Statistical Sciences Division and Center for Nonlinear Studies, Los Alamos National Laboratory, Los Alamos, New Mexico, United States of America; IBM Thomas J. Watson Research Center, United States of America

## Abstract

We quantify the influence of the topology of a transcriptional regulatory network on its ability to process environmental signals. By posing the problem in terms of information theory, we do this without specifying the function performed by the network. Specifically, we study the maximum mutual information between the input (chemical) signal and the output (genetic) response attainable by the network in the context of an analytic model of particle number fluctuations. We perform this analysis for all biochemical circuits, including various feedback loops, that can be built out of 3 chemical species, each under the control of one regulator. We find that a generic network, constrained to low molecule numbers and reasonable response times, can transduce more information than a simple binary switch and, in fact, manages to achieve close to the optimal information transmission fidelity. These high-information solutions are robust to tenfold changes in most of the networks' biochemical parameters; moreover they are easier to achieve in networks containing cycles with an odd number of negative regulators (overall negative feedback) due to their decreased molecular noise (a result which we derive analytically). Finally, we demonstrate that a single circuit can support multiple high-information solutions. These findings suggest a potential resolution of the “cross-talk” phenomenon as well as the previously unexplained observation that transcription factors that undergo proteolysis are more likely to be auto-repressive.

## Introduction

Genetic regulatory networks act as biochemical computing machines in cells, measuring, processing, and integrating inputs from the cellular and extracellular environment and producing appropriate outputs in the form of gene expression. The behavior of these networks is not deterministic; many of the molecules involved in genetic regulation (e.g., DNA, mRNA, transcription factors) are found in low copy numbers, and are thus subject to severe copy number fluctuations. In living cells, the origins and consequences of stochasticity are well-studied [1]–[6]; one can analyze propagation of noise through cellular networks [7] and disambiguate noise from different sources (e.g., *intrinsic* vs. *extrinsic* [8]–[10]). Surprisingly, cells function in the presence of noise remarkably well, often performing close to the physical limits imposed by the discreteness of the signals and the signal processing machinery [11], [12].

From a signal-processing or information-theoretic perspective [13], noise intrinsic to the gene network presents an obstacle for signal transduction and biochemical computation: with too much noise, the information about the state of the environment (the *signal*) may be lost. So strong is the perception that the noise dominates the dynamics of regulatory networks, that the standard model of gene regulation has been that of Boolean logic [14]–[18], effectively implying that, at best, only two distinct states (*on* or *off*) can be resolved in the noisy genetic output. However, one can build stable binary biochemical switches with just tens of copies of a transcription factor molecule [19], which begs the question: Can we do even better with slightly more molecules? That is, is the genetic regulation, indeed, binary?

In fact, many biochemical networks often need to respond (and do respond [20]) with much finer detail than binary logic. As an example, the well-studied *p53* module responds to ionizing radiation in a “digital” manner [21], [22], initiating a number of disparate cellular responses, including cell cycle arrest, apoptosis, and induction of cellular differentiation, among others [23]. The *p53* module (whose elements have been estimated to be at low copy number [22]) must not only transduce a simple binary answer (was there DNA damage or not?), but also more specific information (What was the damage? How severe? What should be done about it?) It is not evident that a few tens of molecules, whose abundance is subject to intrinsic copy number fluctuations, can successfully perform this task. Of note, a series of recent papers studying the effect of single allele loss in various tumor suppressor genes, including *p53*, challenge the classic two-hit model of tumorigenesis [24] by demonstrating dosage-dependent modulation of phenotype (see [25]–[27] and references therein).

The above example is just one of many instances of “cross-talk”–a perplexing phenomenon observed across many cellular signaling systems in which a single noisy biomolecular species, presumably existing in just two states (active/inactive), is able to transmit complex information. Perhaps the most well studied example of cross-talk occurs in the protein signaling mitogen-activated protein kinase (MAPK) pathways. MAPK cascades transduce multiple stimuli from the environment into distinct genetic programs. Many of these signals are transmitted by common components [28], and, for example, the ensuing cross-talk can be exploited by cancer cells to initiate uncontrolled cell growth [29] even in the presence of chemotherapeutic agents targeting individual signaling pathways. In these systems, cells establish specificity by sequestration including cell type, subcellular localization, temporal, or with scaffold proteins [28], [30]–[33]. In some cases sequestration mechanisms are not available and specificity is achieved via signaling kinetics. For example, in mammal pheochromoctyoma cells, ligands triggering distinct programs (proliferate or differentiate) activate the same receptor tyrosine kinase pathway but with different amplitudes [34]. In fact, by increasing or decreasing receptor expression, the wrong program may also be initiated [35], implying that poor control of kinetics may have pathological consequences. Since the number of molecules involved in the decision making can be rather small even for a large number of total molecules [36], a natural question is: What kind of limits does the intrinsic noise put on the specificity of transduction of multiple signals? Or, equivalently: How many binary signals can be transduced by a biochemical network with small number of molecules?

In this paper, we demonstrate that generic small networks under biological constraints can transduce more information than a simple binary switch, often coming close to the *optimal* transmission fidelity, which we calculate numerically and analytically from physical constraints. In particular, this argues against using Boolean descriptions of regulatory or signaling networks and provides a firm justification behind kinetics-based solution for the cross-talk paradox. In our analysis, we choose a general information-theoretic measure of quality of signal transduction by a circuit, thus obviating the problem of requiring prior knowledge of the function of the network [37]–[44], which is obviously network-specific and often unknown, and the related problem that a given network may perform multiple functions [45]–[47]. We also demonstrate that the presence of an odd number of negative regulators in a feedback loop confers an advantage to the circuit in terms of noise regulation and thus information transmission. Finally, we show that the ability to transduce information reliably is insensitive to most large (tenfold) deviations of a network's kinetic parameters.

### Measure of Quality of a Biochemical Computation

To motivate our approach, consider the experimental setup of Guet et al. [15]. Probing experimentally the relationship between structure and function in transcriptional networks, Guet and coworkers built a combinatorial library of 3-gene circuits and looked at the steady-state expression *G* of a reporter gene (GFP), coupled to one of the genes in the circuit, in response to four different chemical inputs *C*, namely two binary states of two different chemicals. The chemicals interacted with the transcription factor proteins in the circuits and affected their ability to regulate transcription of the target genes. Thus the circuits acted as transducers, converting chemical signals into genetic response. Guet et al. found that some topologies could perform different behaviors (that is, behave as different logic gates), while others could achieve only one particular function. Of note, while some circuits responded differently to different inputs, for other circuits, the reporter expression did not depend on the chemical input state. The latter are clearly “broken circuits,” transducing no information about the inputs.

Notice that the responses in [15] appear binary and deterministic due to a two-state discretization (*G* is either *on* or *off*). In fact, the actual number of GFP reporters in each cell clearly is not repeatable due to the stochastic nature of the involved cellular machinery. For this reason, the input-output relation for a circuit should be described not in terms of a deterministic transfer or dose-response function, but by some conditional probability distribution *P*(*g*|*c*)≡*P*(*G* = *g*|*C* = *c*), where *c* stands for particular chemical states, and *g* measures the number of reporter molecules. Then a natural measure of a circuit's quality is the *mutual information* between its inputs and outputs [13]

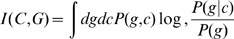
(1)where log is taken always with the base 2, unless noted otherwise. This dimensionless, nonmetric quantity measures in bits the extent to which *C* and *G* are dependent (complete independence implies *P*(*g*,*c*) = *P*(*g*)*P*(*c*), and thus *I*(*C*,*G*) = 0). The mutual information is bounded, 0≤*I*(*C*,*G*)≤min[*H*(*C*), *H*(*G*)], where *H*(*X*) is the entropy, 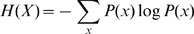. In [15], there were ||*C*|| = 4 possible input states *c*∈{1,2,3,4} = {*c_i_*} and two possible output states, GFP *on* or *off*. For a circuit with a constant *g*, *H*(*G*) = 0, and then *I*(*G*,*C*) = 0. At the other extreme, if the reporter gene is *on* for exactly two of the four equiprobable chemical inputs, then each reporter state has *P* = 1/2, and *I*(*C*,*G*) = 1 bit. Similarly, for multinomial distributions of *g*, the mutual information seamlessly takes into account all possible relations between *g* and *c*.

Note that Eq. (1) avoids any binning or thresholding of data. This makes it possible to make precise the intuition that response states with, say, 10 and 15 molecules of GFP are less different from each other than those with 10 and 150 molecules, even though both pairs can be separated by simple thresholding. Indeed, because of the fluctuations, *P*(*g*|*c*) will be overlapping for the former pair, resulting in small *I*(*C*,*G*), while the overlap will be small in the second case. In fact, one of the central questions of our work is whether in realistic biochemical dynamics, states with small molecule numbers are essentially distinct and thus capable of high-fidelity information transmission.

In a more complicated case where *c* and *g* are both time-dependent, one can generalize Eq. (1) to consider the mutual information between the entire temporal profiles of *c*(*t*) and *g*(*t*), which would treat the biological circuit as a Shannon communication channel [13]. However, such a treatment requires specifying a time-varying input distribution—a subject not yet addressed in the related experiments. We focus instead on Eq. (1), which is equivalent to studying communication properties of biological circuits under an assumption that the signals *c*(*t*) vary slower than the circuits' relaxation times.

A crucial advantage in adopting mutual information as a quality measure is that it can be evaluated independently of the function of the circuit. For steady state responses considered here, the only reasonable way to define a qualitative *function* of the circuit, or to characterize the computation performed by it, is to consider how 〈*g*(*c*)〉 are ordered. As long as all ||*C*|| responses are sufficiently resolved, the mutual information will be ∼log||*C*||, irrespective of the ordering. Thus the mutual information-based circuit quality measure is insensitive to the type of computation performed by the circuit, and is only concerned with whether the computation assigns a different output to each input. Furthermore, due to the *data processing inequality* [13], high *I*(*C*,*G*) is a sufficient condition for a high-quality realization of *any* computational function that depends (stochastically or deterministically) on *P*(*g*,*c*). High *I*(*C*,*G*) is especially important for sensory stages in biochemical signaling, where the same biomolecular species may control responses of many different biochemical modules, requiring high quality information about many different properties of the signal at the same time.

### Proposal

We propose to investigate how the topology of a regulatory circuit affects its computational and information transmission properties, as measured by the steady state signal-response mutual information, Eq. (1). While the results of [15] may be interpreted as revealing that some circuits may perform better than others, this effect can be caused in part by operating at suboptimal kinetic parameters, some of which are biologically easy to adjust to improve the information transmission fidelity. In fact, several identical topologies in [15], differing only in their kinetic properties, performed markedly different functions. To avoid the problem, we study instead the maximum mutual information attainable by the circuit under realistic conditions. Specifically, for a regulatory topology *t*, with a set of kinetic parameters **ϑ** = {ϑ_1_,ϑ_2_,…}, which responds to inducer (input) concentrations *C* = {*c_i_*} = {*c*_1_,*c*_2_,…} with different genetic (output) expression levels *P*(*g*|*c*,**ϑ**), we propose to investigate numerically 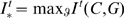.

We emphasize that we do not expect maximization of mutual information to be the sole driving force behind natural selection, and additional constraints (some of which we discuss below) will be important. However, it is also true that, without transmitting information, organisms would not be able to survive. Thus optimization of information transmission may be a relevant selection strategy, at least in some corners of biology (e. g., sensing and morphogenesis [20], [48], [49]). Our work can be viewed as focusing on such corners.

**Figure 1:**
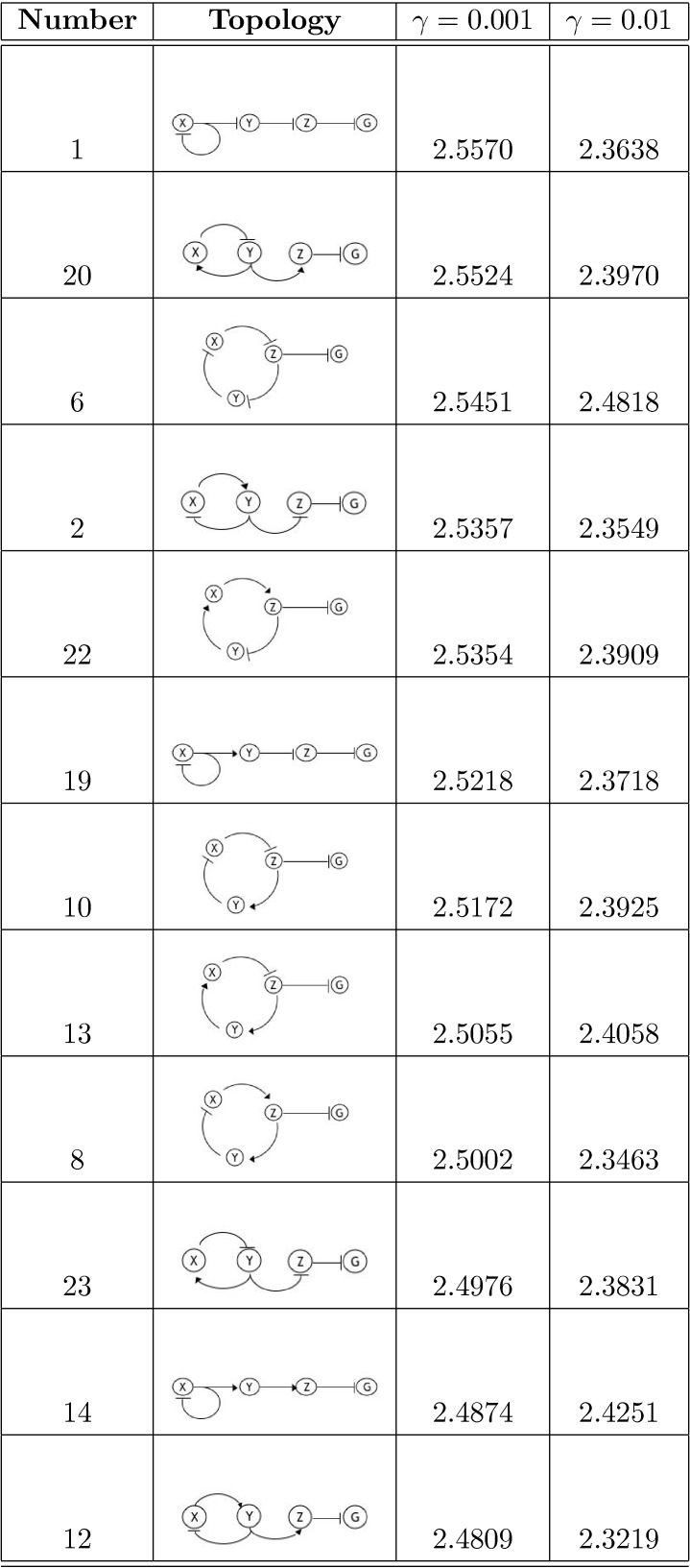
Table of circuits (top 12 by the optimality statistic). Extrapolated average mutual information over range of 25 to 120 molecules at γ = 0.001 and γ = 0.01.

**Figure 2:**
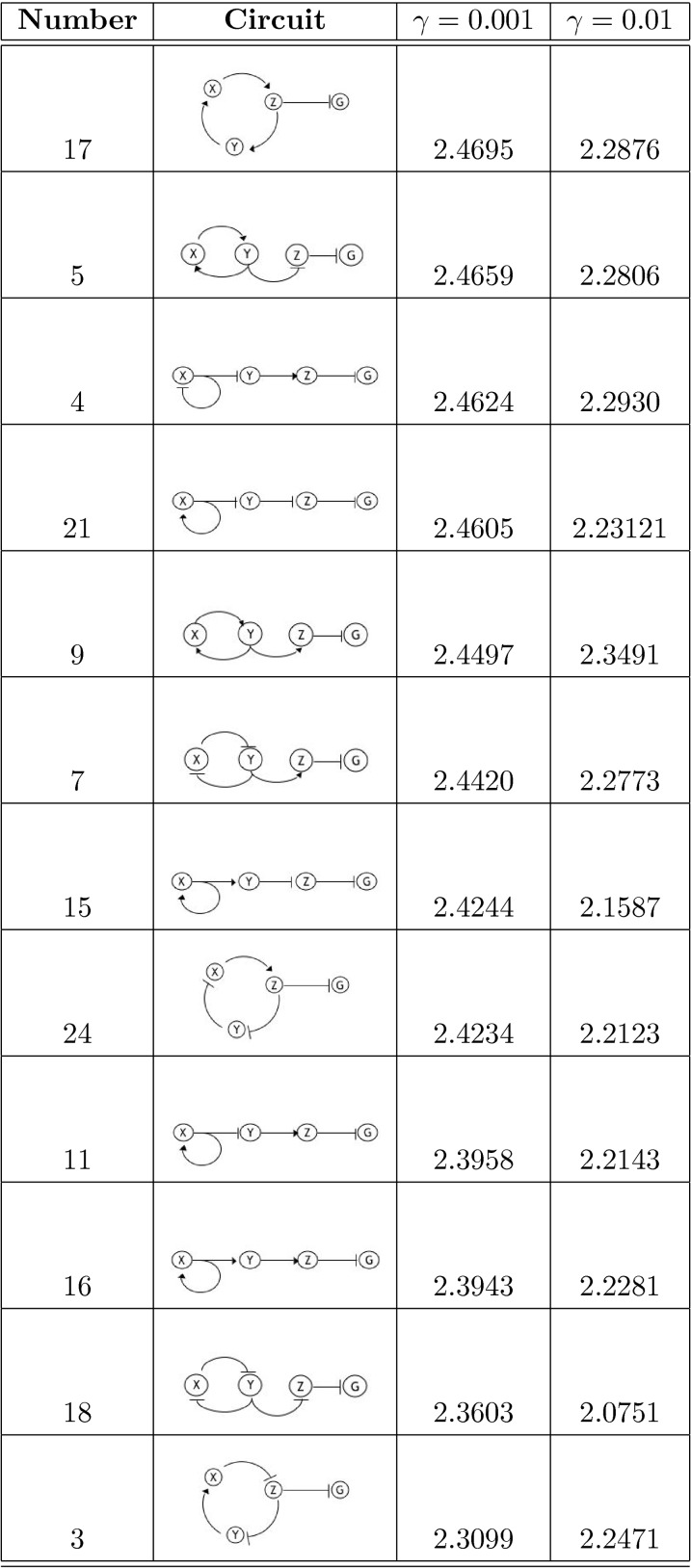
Table of circuits (bottom 12 by the optimality statistic). Extrapolated average mutual information over range of 25 to 120 molecules at γ = 0.001 and γ = 0.01.

As in [15], we limit ourselves to 3-gene topologies where each gene is regulated by exactly one transcription factor (see Figs. 1 and 2 for the list of these topologies, and *Materials and Methods* *: Topologies* for their more detailed description). We measure the output of the circuit in terms of the probability distribution of steady-state expression of the reporter gene, which is always downregulated by another gene denoted *Z* (see Figs. 1 and 2). This limits us to 24 possible circuits, cf. *Materials and Methods* *: Topologies*. The kinetics associated with these topologies are described in *Materials and Methods* *: Model and Parameters.* Note in particular that even though we use the *genetic regulation* terminology throughout the paper, the kinetic model is general enough to account for *protein signaling* and other regulatory mechanisms as well.

For each of the chosen topologies, we need to find stable fixed points of the dynamical systems that describe the circuit, cf. *Materials and Methods* *: Determining Stable Fixed Points*, evaluate the distribution *P*(*g*|*c*) describing fluctuations around these fixed points, estimate the corresponding mutual information *I*(*C*,*G*), and then optimize *I*(*C*,*G*) with respect to the kinetic parameters. Note that all of the parameters of the system that we treat as variable, in fact, can be adjusted by the cell easily over its lifetime by means of many biological mechanisms, cf. *Materials and Methods* *: Model and Parameters*.

Rather than discretizing the reporter output, as in [15], we take into account the actual numbers of the reporter molecules. Assuming mesoscopic (i.e., practically real-valued) copy numbers, we use the linear noise approximation (cf. Sec. *Materials and Methods* *: Linear Noise Approximation* and Text S1) to derive the reporter gene distribution as a sum of Gaussians with means at the stable fixed points. This approximation is common in systems biology literature [50]. Under this assumption, the mutual information between the two random variables, *C* — representing the *discrete* chemical (input) states — and *G* — measuring the *real valued* reporter expression (output) — is
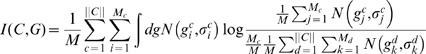
(2)


Here *M* is the total number of fixed point calculations performed for the circuit, and *M_c_* is the number of those done with *C* = *c*; 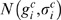 denotes the output response for the *i*'th calculation with *C* = *c*, which is a Gaussian distribution with mean 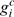 and variance 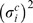. Many calculations at each point in the parameter space, **ϑ**, are needed to explore multiple stable fixed points of the dynamical system (see *Materials and Methods* *: Determining Stable Fixed Points* ). Finally, we choose each chemical state with equal probability, *P*(*c*) = const = 1/||*C*||.

When optimizing Eq. (2) with respect to **ϑ** (see *Materials and Methods* *: Optimization*), we need to consider two computationally trivial (and biologically unrealistic) ways of achieving high *I*(*C*,*G*). First, given discrete *c* and an infinite range of *g*, achieving the upper bound *I*(*C*,*G*) = *H*(*C*) is easy: as the number of molecules of the reporter *g^c^* increases, the magnitude of its fluctuations, as measured by its standard deviation σ*^c^*, grows slower as 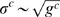, so the responses to all *c*'s can be separated well if we allow for an infinite number of molecules. However, producing many copies of a molecule takes time and energy, both of which are limited. In fact, here we are interested only in solutions that involve low copy numbers, as this is precisely the regime in which gene regulatory networks function. We note also that many apparently deterministic, high copy number systems may actually fall into this regime if the threshold of the system can be overcome with only a few molecules [36], [51]–[54].

Second, and perhaps less obvious, a trivial solution can also be obtained if we allow for multi-scale (*stiff*) systems. For example, if the response time of the reporter τ*_G_* is very large relative to that of the upstream regulators τ*_Z_*, then all of the noisy upstream fluctuations will be filtered [11], [12]: effectively, the reporter measures *N_Z_*τ*_G_*/τ_Z_≫1 molecules of *Z* per reporter's response time (here *N_Z_* is the mean number of *Z* molecules), and fluctuations are small. However, living cells must respond in a timely manner to changes in the environment, so infinite response times are also not biologically relevant.

These observations suggest that our objective function to be maximized requires some biologically reasonable constraints. For this reason, we have investigated many different realizations of the constraints, and, instead of maximizing the mutual information, we chose to maximize the following *constrained mutual information*

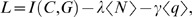
(3)where λ and γ are chosen such that the average number of molecules of all of the components in the system 
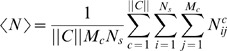
 (where 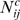 is the average number of molecules of species *i* for fixed point *j* given *C* = *c*, and *N_s_* = 4 is the total number of species in the system) does not exceed: 10^2^, and the average stiffness of the system 
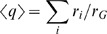
 (where *r_i_* are the decay rates of the transcription factors, and *r_G_* is the decay rate of the reporter) does not exceed: 10^3^.

We note in passing that the copy number and the stiffness constraints are related. Indeed, a standard bandwidth-gain tradeoff in linear signal processing, also studied in a biochemical context [55], suggests that both the copy number and the stiffness can constrained by limiting the energy dissipated by a circuit. However, the actual interplay between the speed and the magnitude of the response with a single constraint is very difficult to pinpoint in our general nonlinear setting, and we chose to utilize the two independent constraints in Eq. (3).

## Results

### Transmitting More Than 1 Bit at Low Copy Number

**Figure 3:**
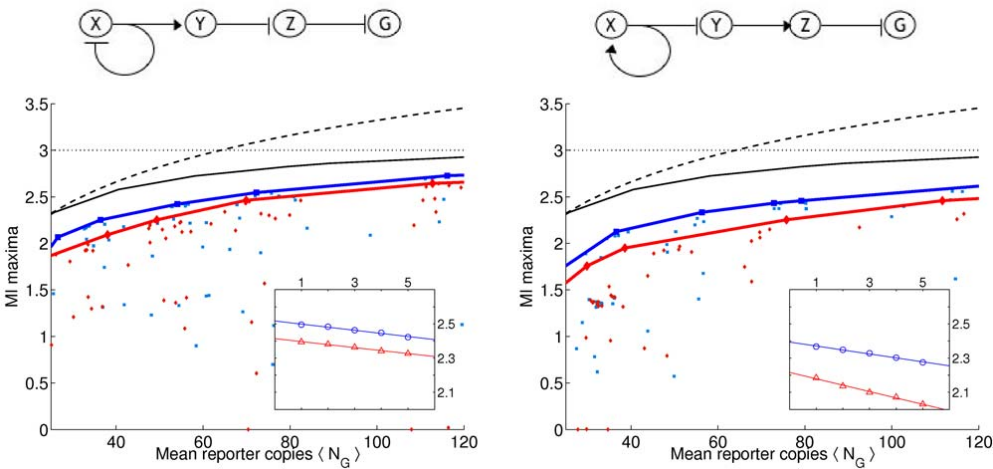
(a) Circuit 19 with an odd number of negative regulators in cycle and (b) Circuit 11 with an even number of negative regulators in cycle. (c) and (d) We ran multiple optimizations ϑ_*_ = argmax_ϑ_ *L*. For each optimization run, we plot the mutual information I_*_ = *I*(*C*,*G*|ϑ_*_) vs. the mean number of molecules of the reporter protein 〈*N_G_*〉. Below 10 copies we saw poor LNA performance (cf. Text S1). Input distribution *p*(*c*) = 1/||*C*|| and ||*C*|| = 8 so that *I*(*C*,*G*)≤*H*(*C*) = 3 bits. Blue squares and red triangles are for γ = 0.001 and γ = 0.01, respectively. The blue and red linearly interpolated lines correspond to the convex hull for each respective γ value. The black solid curve gives the numerically evaluated optimal bound (cf. *Results: Determining Optimal Bounds*) and dashed curve gives analytic bound for any input distribution (cf. *Materials and Methods* *: Maximum Mutual Information for a Fixed Copy Number*). Inset: 〈*I*〉 as a function of the inverse fraction of data included *m* [cf. *Results: (Almost) Optimal Circuits*] in the analysis. Blue and red correspond to two different γ values. Linear regression extrapolated to case of infinite data (y-intercept). The results represent two typical circuits with 1-cycles. Note that here, as in Fig. 4 and Fig. 5, circuits on the left have higher 〈*I*〉 values as well as narrower gaps between the two γ values than circuits on the right.

We tested the ability of each of the 24 different circuits to reliably transduce input signals. For each circuit, we numerically optimized Eq. (3) at different λ and γ. The results of a single optimization thus give us a local maximizer ϑ_*_(λ,γ) of *L*. For each numerically obtained ϑ_*_ we then plot the corresponding mutual information I_*_ [as calculated by Eq. (2)] as a function of the actually observed average number of reporter molecules 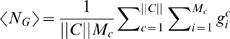. Note that, while I_*_ is a function of the reporter copy number, and we plot I_*_ as this function, the stochasticity of all transcription factors is taken into account in the constraint in *L*, since these are presumably all at low copy number. For example, in Fig. 3 we show the results of multiple maximizations for two typical circuits. Each point on the plot corresponds to a ϑ_*_(λ,γ). The blue squares and the red diamonds correspond to the two different γ values and the solid lines correspond to the “best” solutions which we determine by finding the convex hull of the set of all maxima. Convex hull is used because the noise grows with 〈*N_G_*〉, making an equivalent increase in the number of reporters less potent in transducing more bits at larger 〈*N_G_*〉.

Not surprisingly, as the λ constraint is weakened, and higher molecule numbers are allowed, more information is transduced on average (the blue and red curves always increase monotonically), though some particular solutions do not follow the trend. Similarly, as the γ constraint is increased, and the stiff solutions are constrained, less information is transduced (the red curve is always less than or equal to the blue curve). We report that all 24 topologies can pass more than 1 bit of information with molecule numbers far smaller than 100. In fact, at 25 molecules, most circuits can pass nearly 2 bits of information. In short, generic topologies under biological constraints of response time and molecule numbers *can still transduce more information than a simple binary switch*. Therefore, analyzing such networks in terms of Boolean logic should be questioned.

### Determining Optimal Bounds

To determine how well the circuits performed compared to the optimal behaviour, we first note that all solutions are upper bounded by the entropy of the input distribution, which in our case is *H*(*C*) = 3 bits. Next, recall that the reporter protein, *G*, must be at least subject to its own intrinsic noise, and the variance of this noise must be at least that of a Poisson distribution (*P*(*x*) = exp(−μ)μ*^x^*/*x*!) with mean μ = *g^c^* (since the reporter does not have any feedback) [50]. Given this lower bound and a probability distribution over inputs *C* (in this case, eight equal delta functions), we can numerically calculate an optimal transduction curve. That is, we optimize
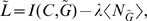
(4)with respect to the mean genetic responses 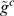, where 
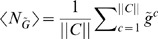
, and 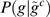 is Poisson. For different values of λ, we can define an optimal curve 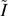 vs. 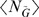, where 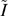 is the mutual information at the maximum of 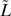. All 24 topologies are upper-bounded by the same resulting curve. Finally we note that 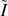 itself is bounded by the channel capacity *I*_0_, which is defined to be the maximum of *I* over all input distributions and can be approximated analytically as in Eq. (27) (see *Materials and Methods* *: Maximum Mutual Information for a Fixed Copy Number*). For 〈*N_G_*〉 = 25 molecules, I_0_≈2.32 bits, and for 〈*N_G_*〉 = 100 molecules, *I*_0_ = 3.32 bits.

### (Almost) Optimal Circuits

We find that all 24 circuits are able to achieve close to the optimal transmission fidelity, implying that they are able to tune the noise from the upstream factors to almost negligible values (see Figs. 3–5 and Text S1, Figs. S1,S2,S3,S4,S5,S6,S7,S8,S9,S10,S11,S12). To quantify how well the circuits perform compared to the optimal bound and to each other, we define the statistic
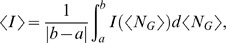
(5)where *I* is the linearly interpolated convex hull and *a* and *b* are set to 25 and 120 molecules, respectively. Note that, for our discrete input distribution, we can upper bound 〈*I*〉≤2.75 bits, where we use the linearly interpolated curve derived numerically 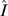, as described in *Results: Determining Optimal Bounds*; similarly, for *any* input distribution we can upper bound 〈*I*〉≤3.03 bits, where we use the analytic approximation *I*_0_ derived in Eq. (27).

**Figure 4 pone-0001077-g004:**
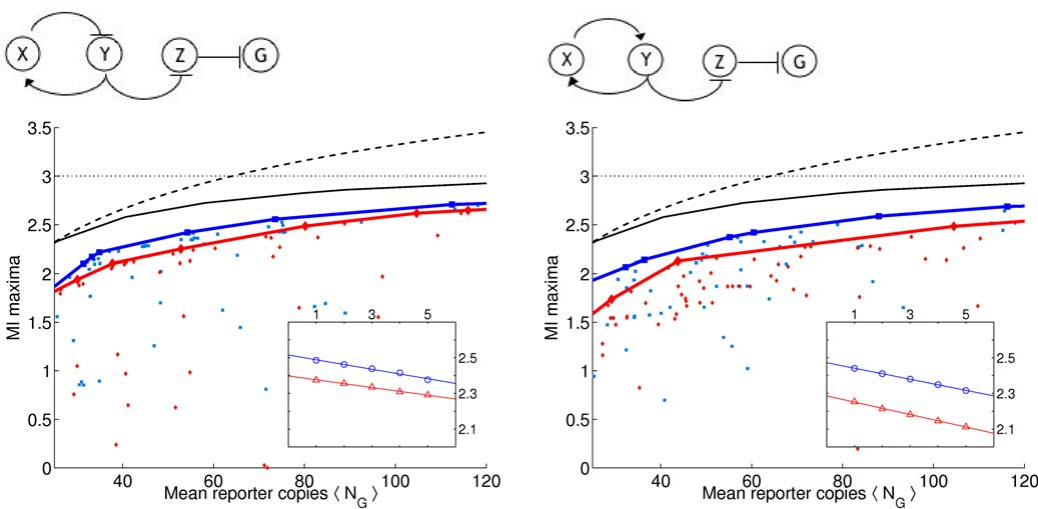
(a) Circuit 23 with an odd number of negative regulators in cycle and (b) Circuit 5 with an even number of negative regulators in cycle. (c) and (d) Same as in Fig. 3 for these two circuits with 2 cycles.

**Figure 5:**
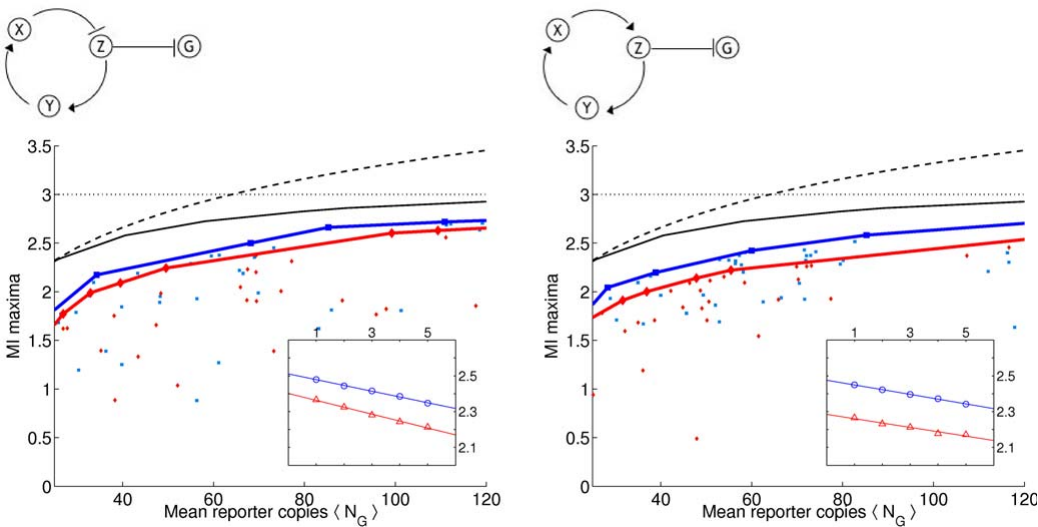
(a) Circuit 13 with an odd number of negative regulators in cycle and (b) Circuit 17 with an even number of negative regulators in cycle. (c) and (d) Same as in Fig. 3 for these two circuits with 3 cycles.

Since the convex hull area can only grow with the number of optimizations we run, there is a bias in our calculated statistic 〈*I*〉. That is, with *k* optimization runs, 〈*I_k_*〉≥〈*I_k_*_−1_〉. We are interested in 〈*I*〉 = 〈*I*_∞_〉, but this is clearly unattainable. Moreover, for different topologies, 〈*I*〉 may be approached with different speeds as a function of *k*, making comparisons between topologies suspect. We use jackknifing to estimate the bias. That is, in the spirit of [56], [57], instead of the total number of optimization runs *N_opt_*, we use only *N_opt_*/*m* of them to calculate 〈*I*〉. Then one can estimate 〈*I*_∞_〉 by fitting
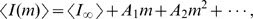
(6)where *A_i_* are some constants. In the insets of Figs. 3–5 we show the dependence of 〈*I*〉 on *m*, the inverse fraction of data included. We see that, for the most part, 〈*I*(*m*)〉 is well fit by a straight line, and contributions from the higher order corrections are insignificant. The results of extrapolating 〈*I*〉 to *m* = 0 for each circuit are reported in Figs. 1 and 2. The average 〈*I*〉 over all circuits is 2.48±0.05 (mean plus/minus standard deviation of the set) and 2.32±0.09 bits for γ = 0.001 and γ = 0.01, respectively. We find that the circuits are within 10% of the optimal transduction capacity of 2.75 bits, as explained above.

### Ranking Circuits

The optimality measure 〈*I*〉 provides a ranking of the topologies (see Figs. 1 and 2). While, strikingly, all of the circuits perform close to the optimal bound, systematic differences still emerge. Consider for example the 8 linear chains with autoregulation (circuits 1, 19, 14, and 4 with negative feedback and circuits 21, 15, 16, and 11 with positive feedback). We note that the negative feedback circuits all have higher 〈*I*〉 values than their positive feedback counterparts. Moreover, the gap between the γ = 0.001 and γ = 0.01 curves is narrower for the negative feedback circuits. That is, even when the stiffness is constrained, these circuits still do well, whereas the positive feedback circuits are more reliant on stiff dynamics. These results are consistent with the findings in [39] that autorepressive circuits can help regulate noise. Interestingly, this trend can be generalized to the circuits with longer cycles as well. For example, we also find that for the 8 circuits with 2-cycles, those that perform best are those that have opposite regulations (one repressive, one activating) rather than two activating or two repressing regulators. For the case of 3-cycles, those circuits with 1 or 3 negative regulators have on average higher values of 〈*I*〉. In Figs. 3–5, we display curves for typical 1-, 2-, and 3-cycles, respectively, with both odd (left column) and even (right column) number of negative regulators.

**Figure 6:**
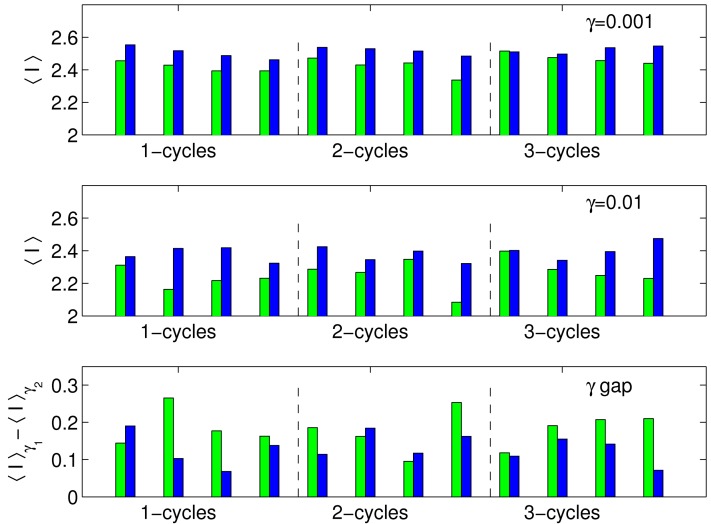
Bar graphs for 〈*I*〉 values for the two classes of circuits: odd (blue) includes circuits with cycles containing an odd number of repressors and even (green) includes circuits with cycles containing an even number of repressors. Top γ_1_ = 0.001, middle γ_2_ = 0.01, and bottom 〈*I*(γ_1_)〉−〈*I*(γ_2_)〉. For all 3 measures, there is a statistically significant difference between the two classes of circuits as calculated by the U Test (top *p* = 0.0002, middle *p* = 0.0003 and bottom *p* = 0.01).

These findings imply that there are some structural constraints that impart small but measurable limitations to the circuit's transduction capacity. In particular, those circuits with an odd number of negative regulators (an overall negative feedback) in their cycles are generally ranked higher than those circuits with an even number of negative regulators (an overall positive feedback), see Figs. 1 and 2. In Fig. 6, we show a bar graph of the values of 〈*I*〉 for the two classes of circuits (odd and even number of negative regulators in the cycle) for different γ values and for different length cycles. The average mutual information for the circuits with an odd number of negative regulators is 2.51±0.03 and 2.39±0.05 for the two γ values, whereas for the circuits with an even number of negative regulators, it is 2.44±0.03 and 2.26±0.05 for the two γ values. Between the two classes, these values are more than one standard deviation apart. To test the significance of this observation, we perform the non-parametric Mann-Whitney U Test [58], [59], which measures the difference in medians between two samples. We find that, for γ = 0.001, *U* = 8, and the *p*-value is 0.0002; and, for γ = 0.01, *U* = 10, and *p*-value is 0.0003. That is, the null hypothesis that the optimality measures for the two classes of circuits (odd and even number of negative regulators, or, alternatively, overall negative and positive feedback) are drawn from the same distribution and, therefore, have the same medians, is highly unlikely.

### Noise analysis

Since circuits containing cycles with an odd number of negative regulators are better signal transducers, we might expect that they are able to control the noise variance better. In fact, using the linear noise approximation (cf. *Materials and Methods* *: Linear Noise Approximation*), we prove this assertion for a generic transcriptional network in *Materials and Methods* *: Network Noise Analysis Using Linear Noise Approximation*. Furthermore, for simple networks, we demonstrate that the overall negative feedback is a necessary and, in one case, even a sufficient condition to achieve sub-Poisson noise (variance less than the mean).

For example, let 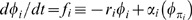 describe the deterministic dynamics of gene *i* (see *Materials and Methods* *: Model and Parameters* for explanation of the notation) and where π*_i_* denotes the set of regulators of *i*. At steady-state 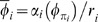. Then, for a 1-cycle where π*_i_* = *i*, Eq. (30) for a species variance reduces to
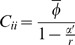
(7)where α′ is the derivative of the gene expression function, and the above is evaluated at the deterministic steady state. In the case of an auto-repression, α′<0, and the variance *C_ii_* is less than the mean [40], [60].

Similarly, Eq. (30) can be reduced for a 2-cycle, *i*, *j* = {1, 2}
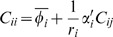
(8)

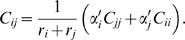
(9)Since *C_ii_*>0, here a necessary (but not sufficient) condition for sub-Poisson noise is 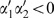.

This analysis (as well as the derivation in *Materials and Methods* *: Network Noise Analysis Using Linear Noise Approximation*) also illustrates that it is easier to obtain smaller variance (and hence larger mutual information), for cycles of shorter length. This is in agreement with [61] where it was found that short cycles are over-represented in a metabolic network, but large cycles occurred less frequently than one would expect given several different possible null models.

### Reliance on Large 〈*q*〉

The “gap” between the two γ curves suggests another statistic to compare the circuits. Presumably, a wide gap implies that the circuit relies on large stiffness 〈*q*〉 to regulate noise. Indeed, for large 〈*q*〉 values, the objective function *L* decreases, though this decrease is moderated by the value of γ such that smaller γ values allow larger 〈*q*〉 values. Stiff solutions have the advantage of allowing the reporter protein to effectively act as a low-pass filter, slowly averaging and responding to fluctuations in the circuit components. A reliance on small values of γ implies that the circuit has more difficulty regulating noise. We therefore expect the circuits with an odd number of negative regulators to have smaller gaps. Consistent with this prediction, while the average gap over all circuit was 0.16±0.05, the average gap for the negative feedback circuits was 0.13±0.04, and for the rest it was 0.18±0.05 (see Fig. 6). The U Test using the gap measure gives *U* = 28 and *p*-value of 0.01, indicating a moderately significant difference.

Evidence from a database of transcription factors in prokaryotes supports the finding that circuits with negative feedback can suppress noise [62]. In *Escherichia coli*, many transcription factors do not undergo active degradation via proteolysis, but are instead only passively degraded via dilution. The half-lives of such proteins are on the order of the division time of the cell, allowing them to respond only slowly to fluctuations in the mRNA concentrations. As is the case of the stiff solutions with high 〈*q*〉 in our circuits, these slowly responding transcription factors have an advantage in noise control [63]. Therefore, we might expect that transcription factors that do not undergo proteolysis will have no auto-repression, or even positive auto-regulation. On the other hand, transcription factors that do undergo proteolysis and cannot, therefore, filter mRNA fluctuations as well would be more likely to require negative auto-regulation.

To test this hypothesis, we analyzed 145 transcription factors of the *E. coli* regulatory network. For each transcription factor we correlated whether the factor is auto-repressive [62] with whether it potentially undergoes proteolysis by noting if the peptide sequence had any known cleavage sites [64]. While the presence of cleavage sites in a protein sequence may mean the protein is more likely to be degraded, it does not necessarily mean that the protein is degraded. Since there is no database containing data about degradation rates of known transcription factors, finding even a moderate correlation between cleavage sites and auto-repression would be interesting. We found that of the 13 transcription factors that are likely to undergo proteolysis, 9 are negative auto-repressors, and out of the 132 transcription factors that are not, 88 are not auto-repressors. A Fisher exact probability test revealed a statistically significant positive association between putative proteolysis and negative feedback (*p*-value 0.013). See Text S1 and Tables S1 and S2 for details.

### Robust, Adaptive Maxima

An important consideration in further assessing the quality of our circuits is the extent to which these high information maxima are *robust* to perturbations in the system. Qualitatively, we define a maximum as robust if, in its vicinity, the cost function *L* does not change significantly in response to perturbation of the parameters *R*,*K*,*a*,*a*_0_, and *s* (see *Materials and Methods* *: Model and Parameters* for parameter definitions). Related, we would also like to consider the ability of our circuits to *adapt*, that is, to change their functional behavior in response to the parameter changes (recall that in our setup a *functional behavior* is defined by the ordering of 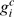). Finally, we would like to understand if a circuit can be robust yet adaptive at the same time.

**Figure 7:**
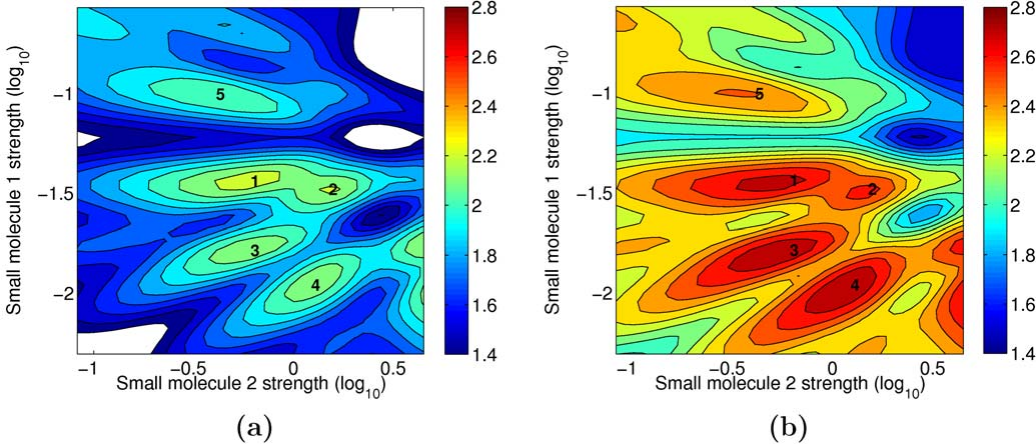
(a) The objective function *L* and (b) the mutual information *I* as a function of the input parameters *s_X_* and *s_Y_* corresponding to the small molecules “strength” on transcription factors *X* and *Y* (cf. *Materials and Methods* *: Model and Parameters*) for circuit 2. The rest of the parameters are held constant for this figure. The five labeled peaks correspond to 5 distinct behaviors or unique signal encodings (cf. Fig. 8 and Table 1).

**Figure 8:**
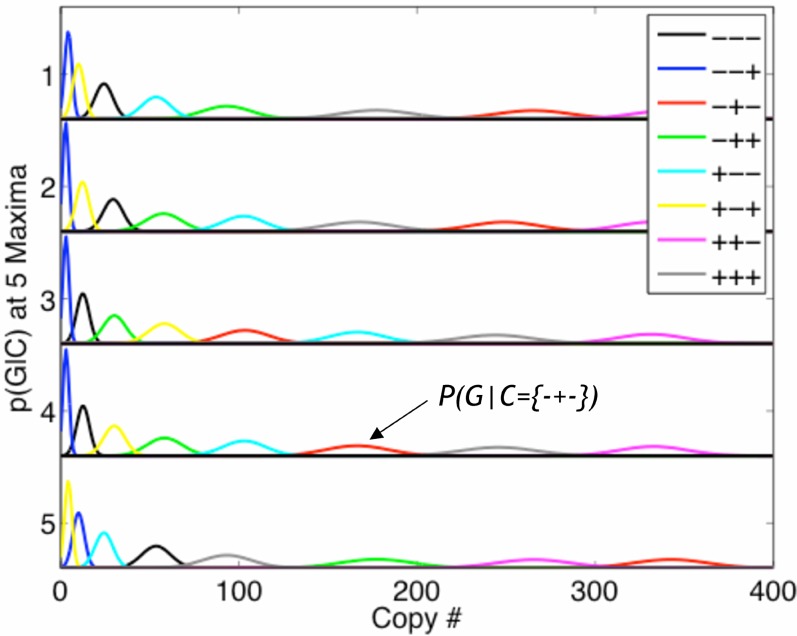
The conditional *p*(*G*|*C*) is plotted for each of the 5 maxima of the constrained information shown in Fig. 7. Colors denote each individual conditional *p*(*G*|*C* = *c*) where *C* takes 8 possible and equally likely, states. Since these are all high information solutions, the individual conditionals are all separated well. Note that at, each maximum, the colors are arranged differently, highlighting the fact that the conditionals are different, and therefore the network behaves differently at each of these high information solutions. The arrangement of these individual conditionals is summarized in Table 1.

While detailed answers to these questions will be reported in a forthcoming publication, here, as a preliminary investigation, we analyzed the functional *L* of circuit 2 near one of its randomly selected maxima. In addition to the original maximum, we found four other distinct nearby peaks as displayed in Fig. 7. The circuit alters its behavior as a result of changes along the 2 displayed dimensions, the strengths of coupling to input 1 (*s_X_*) and to input 2 (*s_Y_*), cf. Eq. (12), so that, at each maximum, the ordering of responses is distinct, and thus the signal is encoded in a different way (i.e., a different computation is performed). The 5 different behaviors or computations are summarized in Table 1 and Fig. 8. Note in Fig. 7 that 4 of the maxima are separated by valleys no deeper than 2.3 bits. In other words, by a change in *s_X_* and *s_Y_* only, the circuit can alter its behavior, while maintaining a high transmission fidelity. In this sense, we consider these maxima to be adaptive.

**Table 1:** Table of behaviors corresponding to the five peaks shown in Fig. 7.

Chemical State	−−−	−−+	−+−	−++	+−−	+−+	++−	+++
**Peak 1**	2	6	1	5	4	8	3	7
**Peak 2**	2	6	4	1	5	8	3	7
**Peak 3**	2	1	4	6	3	5	8	7
**Peak 4**	2	1	6	4	5	3	8	7
**Peak 5**	6	2	5	1	8	4	7	3

Behavior is defined as the ordering of *g^c^*, where *g^c^* is the deterministic steady-state solution for given chemical input *c* and *c* ∈ {(**−−−**), (**−−**+), (**−**+**−**), (**−**++), (+**−−**), (+**−**+), (++−), (+++)}. Each row describes the behavior of the circuit at one of the five maxima.

**Figure 9:**
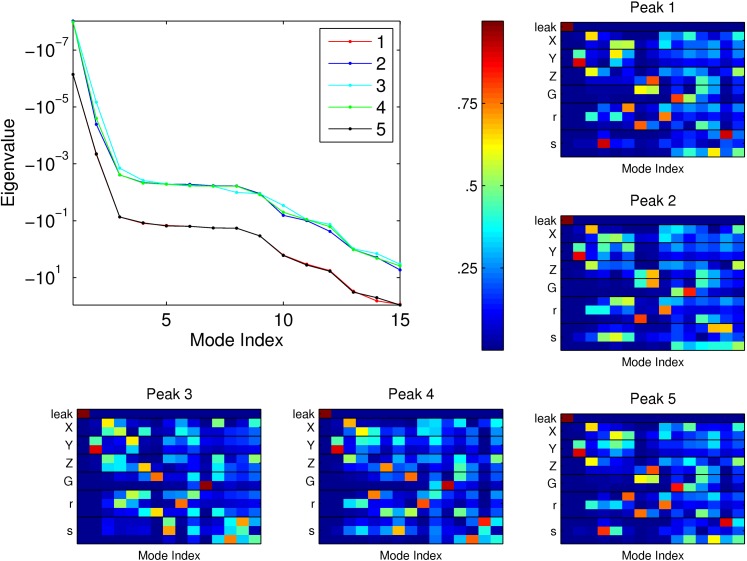
Top-left: Spectra for the numerically calculated Hessian at each of the corresponding 5 peaks labeled in Fig. 7. Soft modes (→0) are directions in which *L* has small curvature; hard modes (→−∞) are directions in which *L* has large curvature. Many eigendirections exhibit small curvature (magnitude of eigenvalue less than 10^−2^ for peaks 2–4 and 10^−1^ for peaks 1 and 5), demonstrating that the maxima are robust to large deviations in parameter space. Colored panels: Magnitude of contribution from each parameter to each eigenvector for each of the five Hessians. Mode index is sorted as in top-left figure (from least curvature to greatest curvature). Row labeled leak corresponds to parameter *a*_0_. Paired rows labeled *X*, *Y*, *Z*, and *G* correspond to the two parameters, *K* and *a*, describing the gene regulation function for each transcription factor (*X*, *Y*, *Z*) and reporter protein (*G*). Rows labeled *r* correspond to the decay rates of each of the 3 transcription factors. Rows labeled *s* correspond to the input parameters modulating the three transcription factors. For all five peaks, the two most soft modes correspond to *a*_0_ and a mixture of *K_Y_*, *a_Y_*, respectively. *s_X_* and *s_Z_* contribute mostly to the hard modes.

To explore sensitivity to parameter perturbations, we next numerically calculated the Hessian at each of the 5 peaks. The Hessian matrix is the square matrix of second partial derivatives of the objective function of *L*. At a maximum of *L*, large negative second derivatives correspond to directions of high curvature and therefore directions in which small perturbations result in large loss in *L*. In Fig. 9, we plot the Hessian eigenvalues along with the corresponding eigenvectors. By treating *L* as locally quadratic near each maximum, we use the Hessian (evaluated with respect to log_10_ of the parameters) to analyze how sensitive the maximum is to deviations in the parameters. For example, for an eigenvalue of −1, moving 10-fold in the corresponding eigendirection would result in a loss of 0.5 for the objective function. Alternatively, an eigenvalue of −0.1 means that we can move 10-fold in that direction, while decreasing the objective function only by 0.05. This should be compared with the typical values near maxima of *L*, *I*∼2 bits. We find that, for most directions for all 5 peaks, eigenvalues magnitudes are less than 0.1. In this sense, we consider these maxima to be robust.

We can identify three different regimes for the eigenvalue spectra: an extremely “soft” regime corresponding to the first two modes, a second soft regime, where the modes 3 to 9 are basically equivalent, and then a third regime (modes 10 through 15), where the eigenvalues become more negative. We note that the spectra for the peaks 1 and 5 overlap almost completely, as do the ones for 2, 3 and 4, and that the latter appear to be more robust (the magnitudes of their eigenvalues are smaller). Interestingly, all five spectra in Fig. 9 are similar, largely due to the fact that the ϑ_*_ are themselves quite similar — that is, the maxima are closely arranged not just in the 2-dimensions displayed in Fig. 7, but over all 15 dimensions. This underscores the circuit's adaptability.

In Fig. 9, we have also displayed the contributions from each parameter to each eigenvector for all 5 peaks. It is clear that the first mode corresponds entirely to the leak parameter, which for all 5 peaks is being driven to 0 as the optimization proceeds. The second mode is also consistent for all 5 peaks, and it corresponds to the parameters *a_Y_* and *K_Y_* (cf. *Materials and Methods* *: Model and Parameters*), governing creation for the transcription factor *Y*. Essentially the range of the gene activation function, *a_Y_*, is driven high while the Michaelis constant *K_Y_* is decreased, so that *Y* is squeezed to low copy numbers, and *G* is an amplified version of its predecessors. This is a reasonable strategy since maximizing the information in the output signal requires that most of the energy spent on building molecules is expended on the reporter protein.

## Discussion

We have presented an information-theoretic, function-independent measure of circuit quality. We have demonstrated that generic small networks can transduce more information than a simple binary switch; moreover, such generic topologies can achieve close to *optimal* transmission fidelity, even under low copy number and fast response time (non-stiff) constraints. Furthermore, high information solutions can be robust to tenfold changes in most parameters.

That such simple stochastic systems can act as good signal transducers suggests a possible explanation for cross-talk, in which multiple ligands trigger the same signaling pathway, and yet reliably produce distinct genetic outputs. Indeed, we have demonstrated that multiple discrete input states can be transduced by the same molecule if the encoding is in molecule numbers even if trivial solutions (high copy number and slow response time) are constrained. To our knowledge, this is the first explanation of how a simple stochastic system can overcome cross-talk that does not invoke the traditional spatial or temporal sequestering argument [30].

It may be possible to correlate properties of the observed optimal information transmission solutions with experiments to investigate to what extent this optimality is essential in biology. For example, a common trend in our circuits was to decrease the decay rate and to increase the average molecule number of the reporter protein or proteins near to it. The slower decay rate allowed temporal filtering, and the copy number distribution allowed to expend the limited resources building reporter molecules which need to encode the entire input signal, rather than wasting them on proteins in the beginning of the circuit. One well-known example of this is in the transcription-translation cascade from DNA to protein. Typically, mRNAs degrade faster than proteins, and their molecular numbers are smaller.

More subtle predictions can be made as well. For example, motivated by the observation that slowly responding regulators have no negative feedback, Rosenfeld et al. [65] have demonstrated that an autorepressive circuit with a strong promoter *causes* faster rise-times. They argue that auto-repression is used as an alternative to increasing the degradation rate. Another explanation for the correlation between fast-response and auto-repression is that fast-responding circuits *require* negative feedback. That is, proteins that undergo degradation are unable to time-average the mRNA fluctuations, and so incorporate other strategies to control noise, in particular, auto-repression. The finding of a significant positive association between autorepression and proteolysis is consistent with both roles for negative feedback. In the case of noise control, proteolysis causes greater fluctuations, which are in turn attentuated with the negative feedback mechanism. In the case of response-time, natural fitness may drive the circuit to evolve simultaneously two different mechanisms to reduce response time, negative feedback and increased degradation.

In their analysis of the phototransduction cascade, Detwiler et al. [55] emphasize that signal processing characteristics of a signaling cascade can be tuned simply by altering the concentrations of proteins, rather than by changing the genetic sequence. That is, the parameters of the system can be optimized on a time scale far shorter than evolution. So too, in our simple circuits, all of the kinetic constants can be regarded as functions of concentrations of proteins extrinsic to the circuit, meaning the parameters may also be tuned on a time scale shorter than the response time of the system. We highlight that circuits supporting multiple distinct maxima should be able to flip between different functional behaviors (that is, exhibit adaptation), and that theoretically the effect can be as rapid as changes in protein concentration. Importantly, based on our findings, such adaptation can still occur without significant loss in transduction capacity along the way.

The fact that the 5 peaks we analyzed collapsed onto two categories of spectra underscores a somewhat paradoxical finding. Namely, the maxima are *robust* in that they can withstand 10-fold perturbations in most of kinetic parameters without a significant loss in transmission fidelity, and yet they are *adaptive* in that the circuit can flip between the different maxima (and different behaviors), again without significant information loss. Intuitively, one might expect a tradeoff between robustness and adaptability. Our findings suggest that the circuits can avert this tradeoff by clustering the maxima in a general region of high transmission fidelity. Certainly a closer and more quantitative analysis of this tradeoff is warranted. For example, it is now established well that a single circuit can support multiple functions [45]. In this vein, one interesting research direction would be to enumerate the functions that a particular circuit can achieve and quantify how easily the circuit can flip between these functions. Whereas our circuits can all be regarded as “optimal” in the sense that they can tune their parameters to transduce the optimal amount of input information, it is evident that subtle distinctions in information processing exist among them. Our setup is well-suited to systematically explore these distinctions (e.g., varying the input distribution, quantifying the mutual information between time-varying input and output signals, and quantifying other statistics of the mutual information landscape rather than optimality).

## Materials and Methods

### Topologies

As in the experimental set-up of Guet et. al [15], we consider 3-node circuits in which genes are regulated by exactly one gene (including the possibility of auto-regulation). This also reduces the assumptions we would otherwise need to make about the dynamics associated with combinatorial regulation. The 3 genes (*X*, *Y*, and *Z*) in each circuit are interconnected by exactly 3 edges. There are only 3 such non-redundant topological structures, which, when we include the possibility of either excitatory or inhibitory interactions, results in 2^3^ = 8 possible configurations per structure, for a total of 24 topologies (see Figs. 1 and 2). The fourth (reporter) gene *G* is always down-regulated by *Z*, as in [15]. Extensions to other topology classes are easily implemented.

### Model and Parameters

The dynamics of transcription and translation have been modeled with a remarkable success for small circuits by avoiding the translation step completely and coupling the genes to each other directly by means of simple rational functions α*_j_* [41], [66], [67]. In general, each of the species **X** = {*X*,*Y*,*Z*,*G*} in the circuit is subject to a degradation and a creation processes
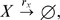
(10)

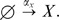
(11)While the dynamics of the circuits is intrinsically stochastic and is always treated as such, it is useful to consider differential equations that govern the evolution of the average chemical concentrations:
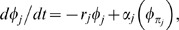
(12)where {*φ*_1_,…,*φ_N_*} is the concentration vector of the *N* chemical components, *r_j_* is the degradation rate of *φ_j_*, and α*_j_* is a production rate that depends on the concentration of a regulator (parent) molecule of *j*, namely π*_j_*. We model the production as a constitutive expression (the *leak*) plus a Hill activation or inhibition,
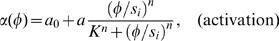
(13)or
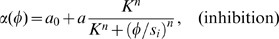
(14)where *a*_0_ describes the leakiness of the promoter, *a* specifies its dynamic range, *K* is the concentration of the regulator at half-saturation (the *Michaelis* constant), *n* is the Hill coefficient, and *s_i_* is the modulating effect of the *i*'th input molecule on the regulator protein (or ratio of the two dissociation constants in the absence and presence of the input molecule). *s_i_* can be modeled equivalently by rescaling *K*. One can think of this as the chemical signal binding to the protein, changing its conformation, and influencing various affinities. This is similar to regulation of the activity of the *lac* repressor by allolactose. For this dynamics, there is no distinction between the protein and the mRNA of a gene species, and we use the terms interchangeably. As in [15], we allow each input to take two binary states (either the input molecule is present or not). We have a total of 3 inputs and 2^3^ input states, and each input modulates the expression of one of the three transcription factors. For a chemical state *c* where an input molecule *i* is not present, we set *s_i_* = 1. We set the units of measurements such that volume Ω of a cell is 1, so that concentration of 1 is equivalent to 1 molecule per cell.

In all, we have 15 parameters:

1. 3 decay rates *r_X_*,*r_Y_*,*r_Z_* corresponding to decay rates for the 3 transcription factors. We set *r_G_* corresponding to a response time of approximately a half hour.
2. 4 *Michaelis* constants *K_X_*,*K_Y_*,*K_Z_*,*K_G_* and 4 range parameters *a_X_*,*a_Y_*,*a_Z_*,*a_G_* describing the regulation function for each component of the circuit.
3. 3 input parameters *s_X_*,*s_Y_*,*s_Z_*, modulating the effect of each input on the 3 transcription factors
4. 1 leak parameter *a*_0_.

For simplicity we assume *n* = 2. This number is consistent with the dimerization typical of bacterial transcription factors. Larger values of *n* would create sharper thresholds in the gene regulation function, though we would not expect qualitatively different results. We have also found similar results for topologies assuming *n* = 1 (results not shown).

Notice that all of these parameters can be easily adjusted by the cell by means of a variety of biological mechanisms, thus validating our proposal to study the dependence of the signal transduction optimized with respect to the parameter values. Below is a non-exhaustive list of such regulatory mechanisms.

1. All protein/mRNA decay rates can be adjusted independently of each other by microRNA expressions or by regulated proteolysis, such as using ubiquitin tagging.
2. Michaelis constants depend on structural properties of proteins and the DNA, as well as on the abundance of the proteins near a DNA binding site compared to the overall protein concentration. Thus they can be adjusted by chromatin rearrangement, or by controlling the nuclear pore transport.
3. Effects of chemical inputs on transcription factors depend on the chemical-protein affinity and on the abundance of the chemicals near the relevant proteins. The former can be changed by modulating chemical-protein binding reaction by means of expression of various enzymes, while the latter can be achieved by controlling transport processes.
4. The leak depends on the concentration of the RNA polymerase, ribosome, as well as the DNA accessibility. All are easy to adjust in a living cell.

### Determining Stable Fixed Points

All of our circuits incorporate some feedback mechanism (e.g., the “feedback dyad” [68]) and, therefore, may have multiple stable steady state solutions. We find these by numerically solving the macroscopic chemical kinetics system (12) describing the circuit using MATLAB's ode15s with the parameters as described in *Materials and Methods* *: Model and Parameters*. We randomly sample different initial conditions for the time-evolution to obtain a set of (almost all) fixed points for each chemical state and each topology. Additionally, since *in vivo* the system will be flipping between different input states, the steady-state solution of one input state is the potential initial condition for the time-evolution of the other inputs. To include these potential initial conditions, we first randomly choose 10 initial conditions for each *c_i_*, and then we take the resulting stable solutions and use them as the initial conditions for each *c_j_*_≠*i*_.

When a time-evolution of the system results in oscillations or chaotic behaviors, we neglect these solutions since, under our assumptions, they will result in multiple genetic outputs corresponding to the same chemical input and hence in a low mutual information. That is, the optimization ends prematurely and we thus disqualify any parameter region which includes these types of behaviors.

### Linear Noise Approximation (LNA)

For excellent reviews and discussions of the Linear Noise technique (also known as the *semiclassical*, fluctuation-dissipation, or linear response approximation), we refer the reader to [50], [69]–[71]. Here we briefly review one particular formulation that simplifies the analysis.

Given a system with volume Ω and *N* different particles, we denote the particle concentrations as *φ* = {*φ*_1_,…,*φ_N_*}, and the copy numbers as *n* = Ω*φ*. The state of the system is defined by *n*, and it changes when an elementary reaction *j*, *j* = 1,…, *R* takes place. When reaction *j* occurs, the copy number *n_i_* changes by *S_ij_*, which is the *N*×*R* stochiometric matrix. Then the evolution of the joint probability distribution *P*(*n*,*t*) is given by the following master equation
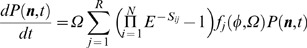
(15)where 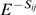 is the *step operator*, which acts by removing *S_ij_* molecules from *n_i_*, and *f_j_* is a rate for *j*.

While this equation is usually mathematically intractable, a Monte Carlo algorithm exists to solve it numerically (the *Gillespie algorithm*) [72], [73]. To generate a particular stochastic trajectory, this method draws random pairs (τ, *e*) from the joint probability density function *P*(τ,*e*|*n*), where τ is the time to the next elementary reaction, and *e* is its index. Multiple trajectories allow to estimate the necessary moments of *P*(*n*,*t*). However, this approach is computationally intensive, and quickly becomes infeasible if one wants to explore multiple system parameterizations, or if *f_j_* span multiple scales. In the latter case, one can often use separation of time scales to achieve adiabatic coarse-graining of dynamics [74].

Alternatively, one can expand the master equation in orders of Ω^−1/2^. Introducing ξ, such that *n_i_* = Ω*φ_i_*+Ω^1/2^ξ*_i_* and treating ξ as continuous, the first two terms in the expansion yield the macroscopic rate and the linear Fokker-Plank equations, respectively:
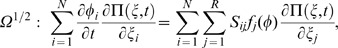
(16)

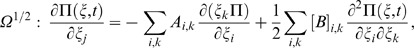
(17)where 
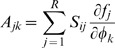
 and 
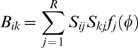
. Note that Eq. (16) is equivalent to and validates Eq. (12).

The steady-state solution of Eq. (17) is a multivariate Gaussian
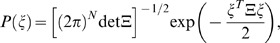
(18)where the covariance matrix Ξ is given by the matrix Lyapunov equation
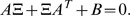
(19)This system is solved using the standard matrix Lyapunov equation solvers (MATLAB's lyap). In order to assess the validity of the linear noise approximation for our system we compared the steady state solutions to multiple Gillespie runs. We found that, even at very low copy numbers (∼10), LNA performed well as measured by the Jensen-Shannon divergence (see Text S1 and Fig. S13 for details). Based on these results, we approximate the steady-state distribution as a sum of multivariate Gaussians with means at the stable fixed points of Eqs. (12, 16) and with covariances as in Eq. (19).

We note that both the LNA and the Gillespie algorithm are derived assuming that the reactions *j* are truly elementary, and therefore have exponential wait times. In our system, a single particle creation, α, encapsulates all processes, starting from the protein-DNA binding and ending with the translation, making the use of the methods questionable (although justification for using “elementary complex” reactions is provided in [70], [75]–[79]). However, the complex nature of the reactions has a comparatively small influence on the low frequency components of the stochastic response [80], which is our focus here. For this reason we believe that approximating terms in Eq. (12) as elementary and using LNA is a less important approximation than merging transcription and translation into a single step. Generalization to LNA with elementary reactions is straight-forward, provided the reaction system is known (which is more complicated).

### Optimization

We employ a simplex optimization (using MATLAB's fminsearch) to maximize *L* = *I*(*G*,*C*)−λ〈*N*〉−γ〈*q*〉 over the log_10_ of the 15 parameters where λ and γ are chosen to accommodate biologically relevant molecule numbers and stiffness. For example, for an average of approximately 100 molecules for each transcription factor and a stiffness of order 1000, we choose λ = 0.01 and γ = 0.001. To explore the parameter space for each topology, we uniformly randomly select biologically relevant starting points (protein half-life near 10 minutes, promoter leakiness near 0.01 proteins/sec, promoter range near 10 proteins/sec, regulator at half-saturation near 100 proteins/sec, and input molecule modulation of regulator near 2). To make the search for maxima more efficient we only maximize random points that start already above a certain threshold (*L*≥0).

### Maximum Mutual Information for a Fixed Copy Number

Suppose a molecular species *G* with concentration *g*, ∫*dgP*(*g*)*g* = 〈*N_G_*〉 is used as a reporter species for a cascade of biochemical computations, so that the species is not allowed to participate in any feedback loops. Then its stochasticity is limited from below by a Poisson noise. That is, if *g^c^* is the deterministic value of *g* produced by some biochemical reaction kinetics, and *g^c^*≫1, then
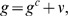
(20)

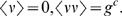
(21)Furthermore, *g^c^* itself is distributed probabilistically according to *P*(*g^c^*), 
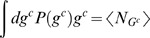
, due to stochasticity of inputs to and of the internal dynamics of the biochemical system. We are interested in the maximum number of bits that can be transmitted reliably by this reporter species (that is, its channel capacity) at fixed 〈*N_G_*〉.

Intuitively, the noise in this system is 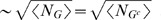, so the number of distinguishable states of the reporter is also 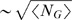, and one should be able to transmit about 1/2 log_2_〈*N_G_*〉 bits reliably. This argument has been used extensively (e.g., [55]). However, it fails (a) to establish the correct constant of proportionality in front of the number of distinguishable states and (b) to take into the account the *g^c^* dependence of the noise variance (which leads to a higher resolution at smaller *g^c^*). Both of these effects are likely to contribute only *O*(1) bits to the channel capacity, but, for 〈*N_G_*〉<100 considered in this work, this might be an important correction. We are unaware of a prior derivation of the channel capacity for this system up to *o*(1), and we present it here.

We write:
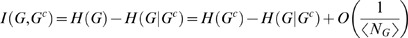
(22)

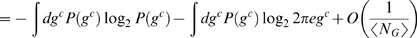
(23)Eq. (22) is valid if var(*G*|*G^c^*)∼〈*N_G_*〉 = var(*G^c^*)∼〈*N_G_*〉^2^, and Eq. (23) holds for a Poisson noise in the reporter.

To find the channel capacity of the reporter species, we maximize *I*(*G*,*G^c^*) with respect to *P*(*g^c^*) subject to
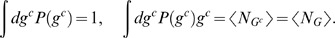
(24)This results in
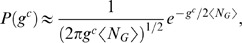
(25)where ≈ is due to the approximation involved in replacing *H*(*G*) by *H*(*G^c^*). Plugging *P*(*G^c^*) into the equation for *I*, we get the channel capacity
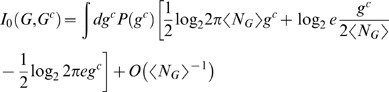
(26)

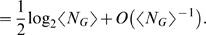
(27)


Thus, for the optimal distribution of inputs, as in Eq. (25), the naive estimate of *I*_0_ = 1/2 log_2_〈*N_G_*〉 for a biochemical reporter is correct up to terms non-vanishing with 〈*N_G_*〉^−1^. For the distribution of inputs analyzed in this work (up to 8 discrete input states), the maximum possible *I*(*G*,*G^c^*) is clearly less than this channel capacity. One can obtain the maximum information for such input distributions by numerical optimization of *I* with respect to the values of the *g^c^* input states, assuming a Poisson distribution of *g* around *g^c^*. This maximum mutual information for 8 input states, as well as the channel capacity, Eq. (27), is shown in, for example, Fig. 3.

### Network Noise Analysis Using Linear Noise Approximation

Consider a regulatory network of *N* transcription factors indexed by *i* ∈ {1,2,…,*N*}. The average concentrations in the system evolve as
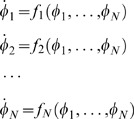
where *φ_i_* is the concentration of the *i*'th transcription factor. Let ***n*** = Ω***φ*** be the vector of molecule copy numbers with volume Ω. Using the linear noise approximation [16], we can calculate the covariance matrix *C* = 〈(**n**−〈**n**〉)(**n**−〈**n**〉)*^T^*〉 = ΞΩ by solving Eq. (19):
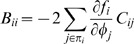
(28)

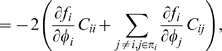
(29)

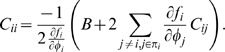
(30)This suggests that the topology or structure of the network can also play a role in controlling noise. Specifically, the variance of the *i*'th transcription factor *C_ii_* can be reduced by decreasing the product 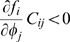, where *j*∈π*_i_*.

The covariance *C_ij_* is a more complicated function of the other covariances:
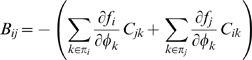
(31)

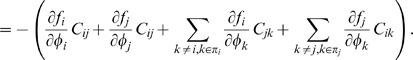
(32)

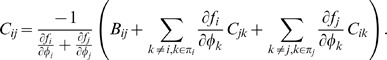
(33)If *j*∈π*_i_*, then, from Eq. (33), we see that *C_ij_* is a function of the covariances between *i* and the regulators of its regulators (*C_ik_*, where *k*∈π*_j_*, and *j*∈π*_i_*). We can write these covariances in turn as functions of covariances between *i* and the regulators of regulators of regulators of *i*, and so on. This implies a recursion, which will end when we either reach a regulator that has no other regulators or, in the case of a cycle, we reach *i* again.

In the latter case, the recursion will end back with *C_ii_*, and the last term in Eq. (33) will have the form
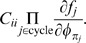
(34)Since *C_ii_*≥0, this implies that one way to reduce *C_ij_* (and hence *C_ii_* itself) is to have the product in Eq. (34) that is *negative*. Crucially, the only way to achieve this is if the cycle contains an odd number of negative regulators.

### Some Simple Examples of Sub-Poisson Noise

The transcription factors in the network may participate in various feedback loops. In some cases, this allows the usual Poisson noise lower bound to be overcome, resulting in a sub-Poisson noise (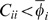). Below we give some simple examples for 1-,2-,and 3-cycles.

The set-up of [15], which we use in this work, simplifies the analysis since we only consider one promoter transcription factors, so that 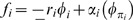, where π*_i_* includes just one gene. In steady state, 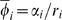. Finally, all of our reactions are enzymatic, so the diffusion matrix *B* will only have diagonal nonzero elements. Then, since 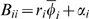, we use the expression for 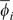 to find 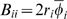.

#### Auto-repression

For the auto-repressive case there are no covariance terms and 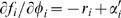, so we can rewrite
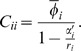
(35)Auto-repression implies 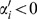. Thus 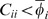, resulting in a sub-Poisson noise.

A similar derivation using the linear noise approximation is given for regulated degradation in [81] and regulated synthesis in [82].

#### A 2-cycle

In this case, π*_i_* = *i*−1, and π*_i_*_−1_ = *i*. Assuming no auto-regulation, let 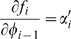 and 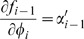. Now we write,
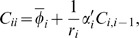
(36)

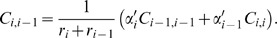
(37)


To reduce *C_ii_* we can reduce the magnitude of *C_i_*_,*i*−1_. One way to achieve this is to have opposite signs for 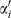 and 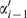. Moreover, the sub-Poisson noise is possible if 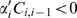, which is possible only if 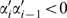. Thus the presence of a negative and positive regulator in a 2-cycle is a necessary, but not sufficient condition for achieving sub-Poisson noise. For sufficiency, we also need 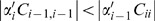.

#### A 3-cycle

In this case, π*_i_* = *i*−1, π*_i_*_−1_ = *i*−2, and π*_i_*_−2_ = *i*. The variance equation stays the same
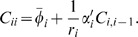
(38)However, now we have
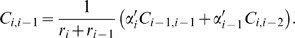
(39)and
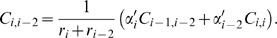
(40)Combining the above into a single expression for *C_ii_*, we have
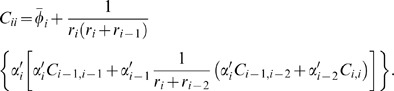
(41)The last term gives us a product of the derivatives, 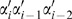. If this product is negative (that is, if we have an odd number of repressors in the cycle) then we can reduce the overall magnitude of the variance *C_ii_*. Note here that we have two extra terms in the variance. One, 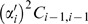, is always positive, while the other can be of either sign. Thus the overall negative regulation is not a guarantee of a sub-Poisson noise in this case.

Ultimately, noise regulation can be improved with cycles with odd number of negative regulators. However, as cycles get larger and the network becomes more complex, the achievability of sub-Poisson noise becomes more limited. This may be related to the observation that, whereas small cycles are over-represented in a metabolic network, large cycles occur less frequently than one would expect given several different possible null models [61].

## Supporting Information

Text S1 Supplementary Text
(0.06 MB DOC)

Figure S1 Mutual Information *I* versus the mean reporter copy number 〈*N_G_*〉 for circuits 1 and 2. Insets: Extrapolated 〈*I*〉 versus the inverse data fraction m as described in the Main Article.
(0.47 MB TIF)

Figure S2 Mutual Information *I* versus the mean reporter copy number 〈*N_G_*〉 for circuits 3 and 4. Insets: Extrapolated 〈*I*〉 versus the inverse data fraction m as described in the Main Article.
(0.47 MB TIF)

Figure S3 Mutual Information *I* versus the mean reporter copy number 〈*N_G_*〉 for circuits 5 and 6. Insets: Extrapolated 〈*I*〉 versus the inverse data fraction m as described in the Main Article.
(0.48 MB TIF)

Figure S4 Mutual Information *I* versus the mean reporter copy number 〈*N_G_*〉 for circuits 7 and 8. Insets: Extrapolated 〈*I*〉 versus the inverse data fraction m as described in the Main Article.
(0.45 MB TIF)

Figure S5 Mutual Information *I* versus the mean reporter copy number 〈*N_G_*〉 for circuits 9 and 10. Insets: Extrapolated 〈*I*〉 versus the inverse data fraction m as described in the Main Article.
(0.46 MB TIF)

Figure S6 Mutual Information *I* versus the mean reporter copy number 〈*N_G_*〉 for circuits 11 and 12. Insets: Extrapolated 〈*I*〉 versus the inverse data fraction m as described in the Main Article.
(0.45 MB TIF)

Figure S7 Mutual Information *I* versus the mean reporter copy number 〈*N_G_*〉 for circuits 13 and 14. Insets: Extrapolated 〈*I*〉 versus the inverse data fraction m as described in the Main Article.
(0.44 MB TIF)

Figure S8 Mutual Information *I* versus the mean reporter copy number 〈*N_G_*〉 for circuits 15 and 16. Insets: Extrapolated 〈*I*〉 versus the inverse data fraction m as described in the Main Article.
(0.43 MB TIF)

Figure S9 Mutual Information *I* versus the mean reporter copy number 〈*N_G_*〉 for circuits 17 and 18. Insets: Extrapolated 〈*I*〉 versus the inverse data fraction m as described in the Main Article.
(0.47 MB TIF)

Figure S10 Mutual Information *I* versus the mean reporter copy number 〈*N_G_*〉 for circuits 19 and 20. Insets: Extrapolated 〈*I*〉 versus the inverse data fraction m as described in the Main Article.
(0.45 MB TIF)

Figure S11 Mutual Information *I* versus the mean reporter copy number 〈*N_G_*〉 for circuits 21 and 22. Insets: Extrapolated 〈*I*〉 versus the inverse data fraction m as described in the Main Article.
(0.46 MB TIF)

Figure S12 Mutual Information *I* versus the mean reporter copy number 〈*N_G_*〉 for circuits 23 and 24. Insets: Extrapolated 〈*I*〉 versus the inverse data fraction m as described in the Main Article.
(0.49 MB TIF)

Figure S13 Jensen-Shannon divergence *JS*_Π_ between distributions obtained by the linear noise approximation and the Gillespie algorithm for multiple circuits and multiple parameterizations plotted as a function of mean copy number. At *JS*_Π_ = 0, the distributions are identical. There appears to be a sharp threshold at 10 molecules, below which the linear noise approximation does poorly, but above which, the linear noise approximation does well.
(0.51 MB TIF)

Table S1 Comparison of presence or absence of proteolysis to presence or absence of negative auto-regulation. Fisher exact probability test reveals signficant (p = 0.013) positive association. This confirms our prediction that transcription factors which undergo proteolysis, and therefore have faster response times, are less able to regulate noise using the temporal filtering, and they require the presence of negative auto-regulation to help control the noise.
(0.03 MB XLS)

Table S2 145 transcription factors in E. coli gene regulatory network as obtained from RegulonDB [4]. Number of cleavage sites is based on MEROPS [5] database and autoregulation (repression = −1, excitation = +1, none = 0) is based on data from [4].
(0.05 MB XLS)
