# Supplementary material for: Optimal Signal Processing in Small Stochastic Biochemical Networks
Source: PLoS One. 2007 Oct 24;2(10):e1077. doi: 10.1371/journal.pone.0001077 (PMC2034356; doi:10.1371/journal.pone.0001077)
Supplement: Text S1 — Supplementary Text (0.06 MB DOC) [file pone.0001077.s001.doc]

# Supplementary Materials S1 for “Optimal signal processing in small stochastic biochemical networks”

Etay Ziv Ilya Nemenman, Chris H. Wiggins,

College of Physicians & Surgeons,

Department of Biomedical Engineering,

Department of Applied Physics and Applied Mathematics,

Center for Computational Biology and Bioinformatics;

Columbia University, New York, NY 10027, USA

Computer, Computational and Statistical Sciences Division and

Center for Nonlinear Studies

Los Alamos National Laboratory, Los Alamos, NM 87545, USA

## *I*(*C*,*G*) vs Curves

We ran multiple optimizations . For each optimization run, we plot the mutual information vs. the mean number of molecules of the reporter protein . Input distribution *p*(*c*)=1/||*C*|| and ||*C*||=8 so that *I*(*C*,*G*) ≤ *H*(*C*) = 3 bits. Blue squares and red triangles are for *γ* = 0.001 and *γ* = 0.01, respectively. The blue and red linearly interpolated lines correspond to the convex hull for each respective *γ* value. The black solid curve gives the numerically evaluated optimal bound and the dashed curve gives analytic bound for any input distribution (cf. Main Article). Inset: 〈*I*〉 as a function of a fraction of data *m* included in the analysis, as in the Main Article. Blue and red again correspond to two different *γ* values. Figs. S1-S12 present results for all 24 circuits.

## Linear Noise Approximation Performance

Here we quantify the linear noise approximation accuracy as a function of the mean molecule numbers in the system. We approximate the steady-state probability distribution using the linear noise approximation (LNA) and compare this to the steady-state distribution obtained with multiple trajectories of the Gillespie algorithm. As a measure of the similarity between the two probability distributions, we use the symmetric *Jensen-Shannon divergence*

(1)

where is the Kullback-Leibler divergence, and we set . We note that , and iff .

We calculated for multiple circuits and multiple parameterizations and found excellent consistency between LNA and Gillespie (see Fig. S13). Below 10 molecules, the two distributions were easily distinguishable, but above 10 molecules we consistently found them similar.

## Positive Association between Proteolysis and Negative Auto-regulation in *E. coli* Transcription Factors

Negative auto-regulation is a “motif” that appears more often than expected (given a particular null model) in both prokaryotic and eukaryotic transcription networks [1]. Becksei and Serrano demonstrate that auto-repressive transcription factors can reduce intrinsic fluctuations and hypothesize that this is one reason that such a motif is ubiquitous in such networks [2]. Meanwhile, Rosenfeld et al. show that this same motif can also speed the response time of the network [3]. They argue that negative feedback is an alternative (and better) strategy to accomplish fast response times than increasing degradation rate and cite as evidence the fact that systems without auto-repression tend to respond slower.

We note that faster response times come at the cost of potentially noisier outputs, and hence poorer transmission fidelity, since the output protein cannot filter out the fluctuations from its regulator or the mRNA molecule precursor. If negative auto-regulation is indeed utilized in a noise-reducing role, one might expect to find a positive association between auto-repressive transcription factors, and transcription factors which undergo proteolysis since degraded proteins will have faster response times. In other words, we hypothesize that negative auto-regulation is utilized to offset the increased stochasticity of fast-responding transcription factors.

To test this hypothesis, we looked at 145 transcription factors from *E. coli* gene regulatory network as obtained from RegulonDB [4]. We used the number of cleavage sites (as determined by MEROPS [5] database) as a proxy for presence or absence of proteolysis. Autoregulation (repression =-1, excitation =+1, none =0) is based on data obtained from [4]. In Table S2, we list our findings and, in Table S1, we summarize the results. We found there to be a total of 13 transcription factors that undergo proteolysis (132 that do not) and 53 transcription factors that are auto-repressive (and 92 that are not). Note that in this analysis we categorize no feedback and positive feedback as the same (no auto-repression). A Fisher exact probability test reveals a significant positive association between proteolysis and auto-repression (*p* = 0.013). Whereas in [3] the association between slow response and no negative feedback was attributed to the causal relationship (fast response is caused by negative feedback), we argue that the association between no proteolysis and no negative feedback may also be due to another causal relationship: fast response is caused by proteolysis which in turn *requires* negative feedback to control noise. However, we note that this does not imply that negative feedback is not also used as a mechanism to shorten rise-time. For example, a circuit may have evolved both negative feedback and increased degradation independently to decrease response-time, in which case the same correlation between proteolysis and auto-repression might be observed.

## References

[1] R Milo, S. Shen-Orr, S Itzkovitz, N Kashtan, U Alon (2002) Simple building blocks of complex networks. *Science*, 298:824–7.

[2] A Becskei, L Serrano (2000) Engineering stability in gene networks by autoregulation. *Nature*, 405:590.

[3] N Rosenfeld, MB Elowitz, U Alon (2002) Negative autoregulation speeds the response times of transcription networks. *J. Mol. Biol.*, 323:785–793.

[4] H Salgado, S Gama-Castro, M Peralta-Gil, E Diaz-Peredo, F Sanchez-Solano, et al. (2006) RegulonDB (version 5.5): Escherichia coli K-12 transcriptional regulatory network, operon organization, and growth conditions. *Nucl. Acids Res.*, 34:D394–7.

[5] ND Rawlings, FR Morton, AJ Barrett (2006) MEROPS: the peptidase database. *Nucl. Acids Res.*, 34:D270–2.
