# Supplementary figures and images for: Optimal Signal Processing in Small Stochastic Biochemical Networks
Source: PLoS One. 2007 Oct 24;2(10):e1077. doi: 10.1371/journal.pone.0001077 (PMC2034356; doi:10.1371/journal.pone.0001077)

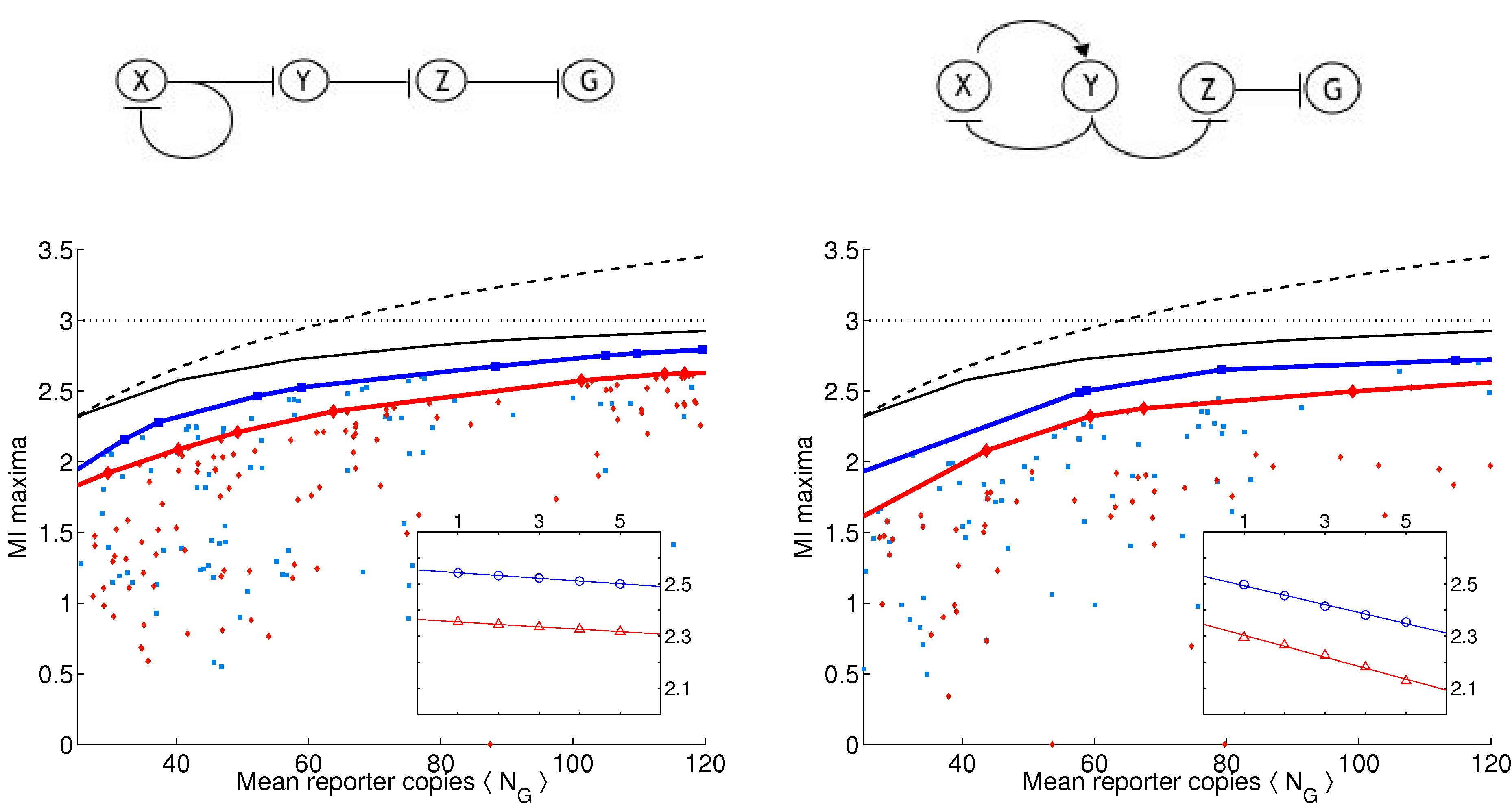

Supplement: Figure S1 — Mutual Information I versus the mean reporter copy number 〈NG〉 for circuits 1 and 2. Insets: Extrapolated 〈I〉 versus the inverse data fraction m as described in the Main Article. (0.47 MB TIF) [file pone.0001077.s002.tif]

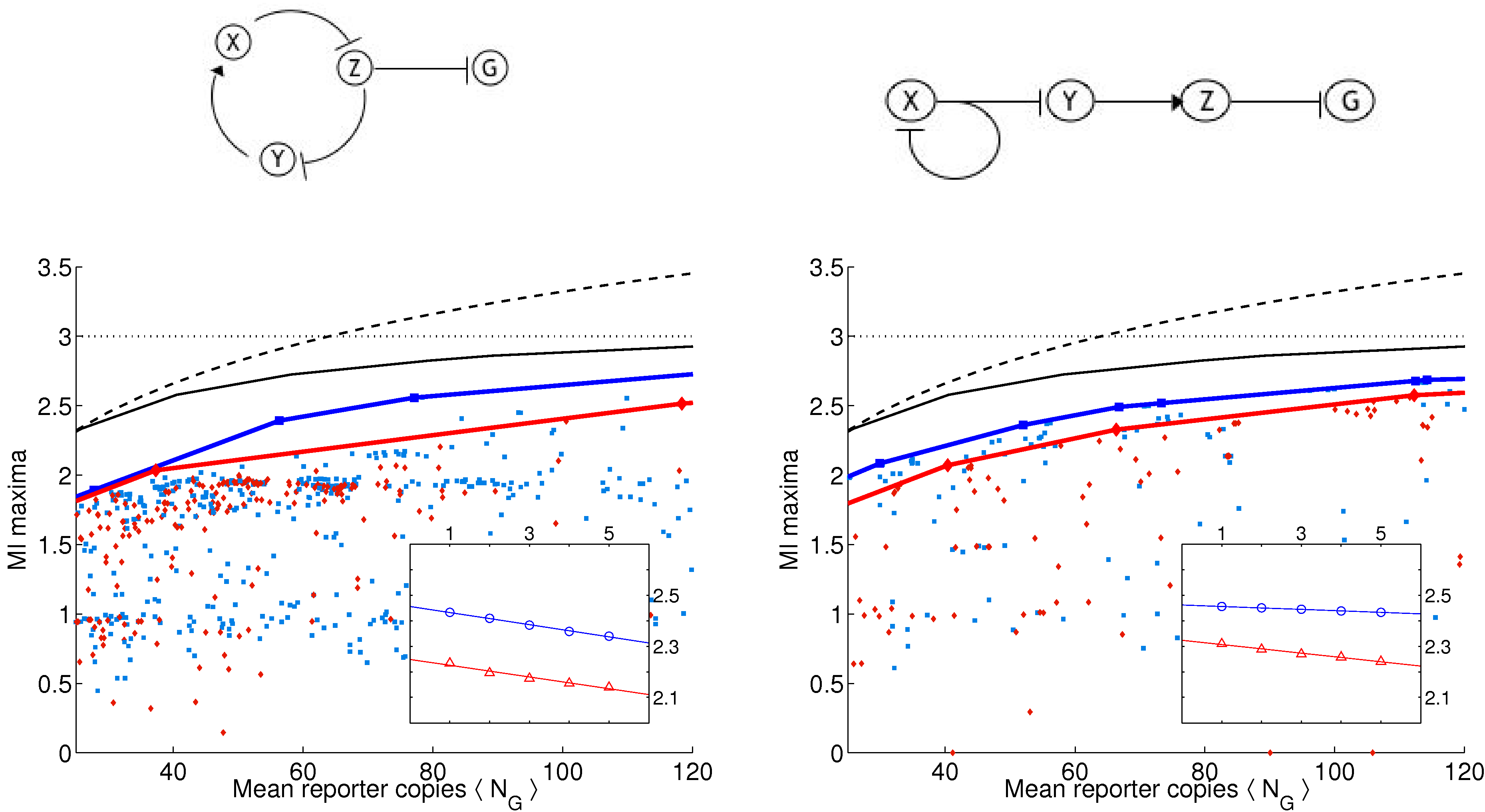

Supplement: Figure S2 — Mutual Information I versus the mean reporter copy number 〈NG〉 for circuits 3 and 4. Insets: Extrapolated 〈I〉 versus the inverse data fraction m as described in the Main Article. (0.47 MB TIF) [file pone.0001077.s003.tif]

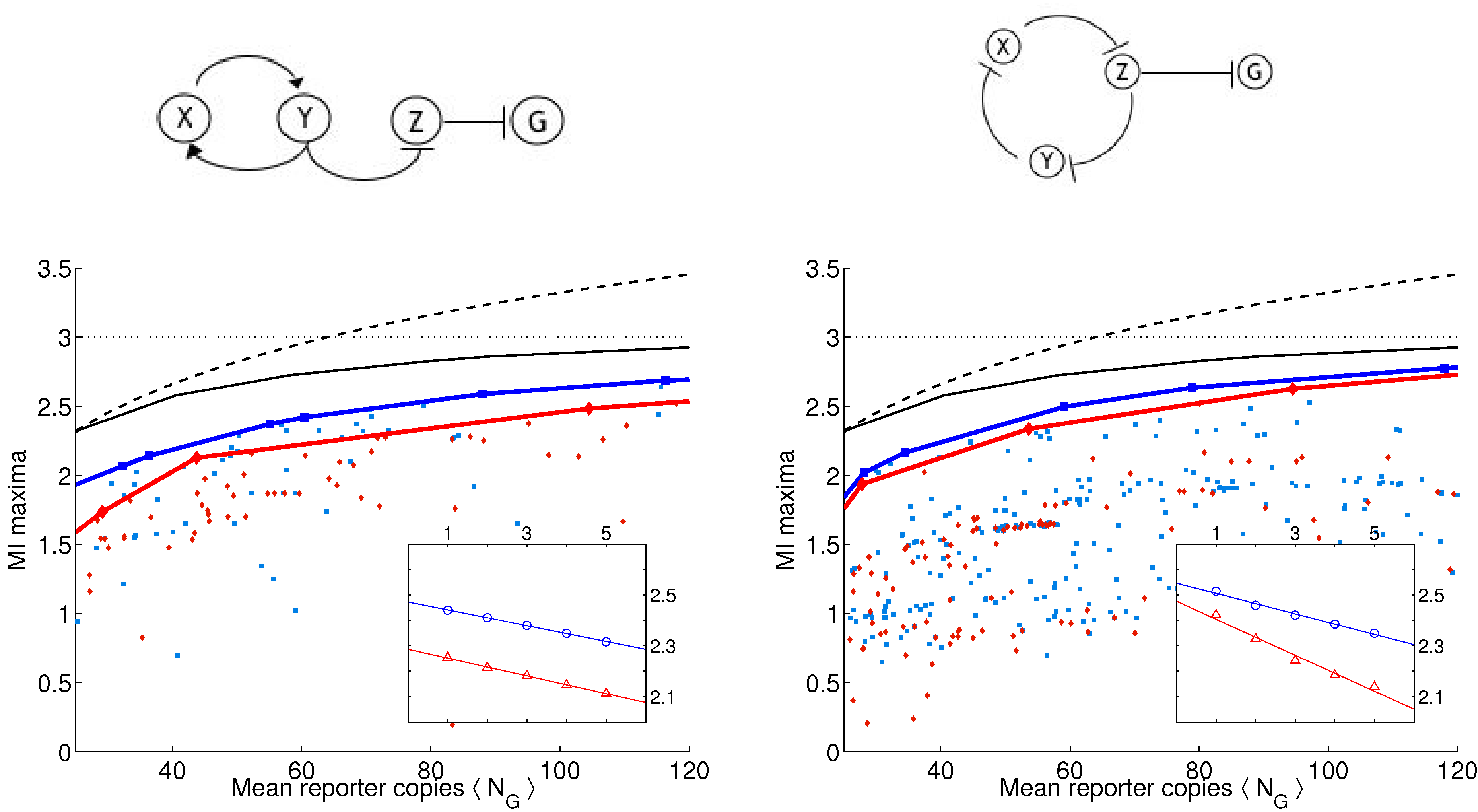

Supplement: Figure S3 — Mutual Information I versus the mean reporter copy number 〈NG〉 for circuits 5 and 6. Insets: Extrapolated 〈I〉 versus the inverse data fraction m as described in the Main Article. (0.48 MB TIF) [file pone.0001077.s004.tif]

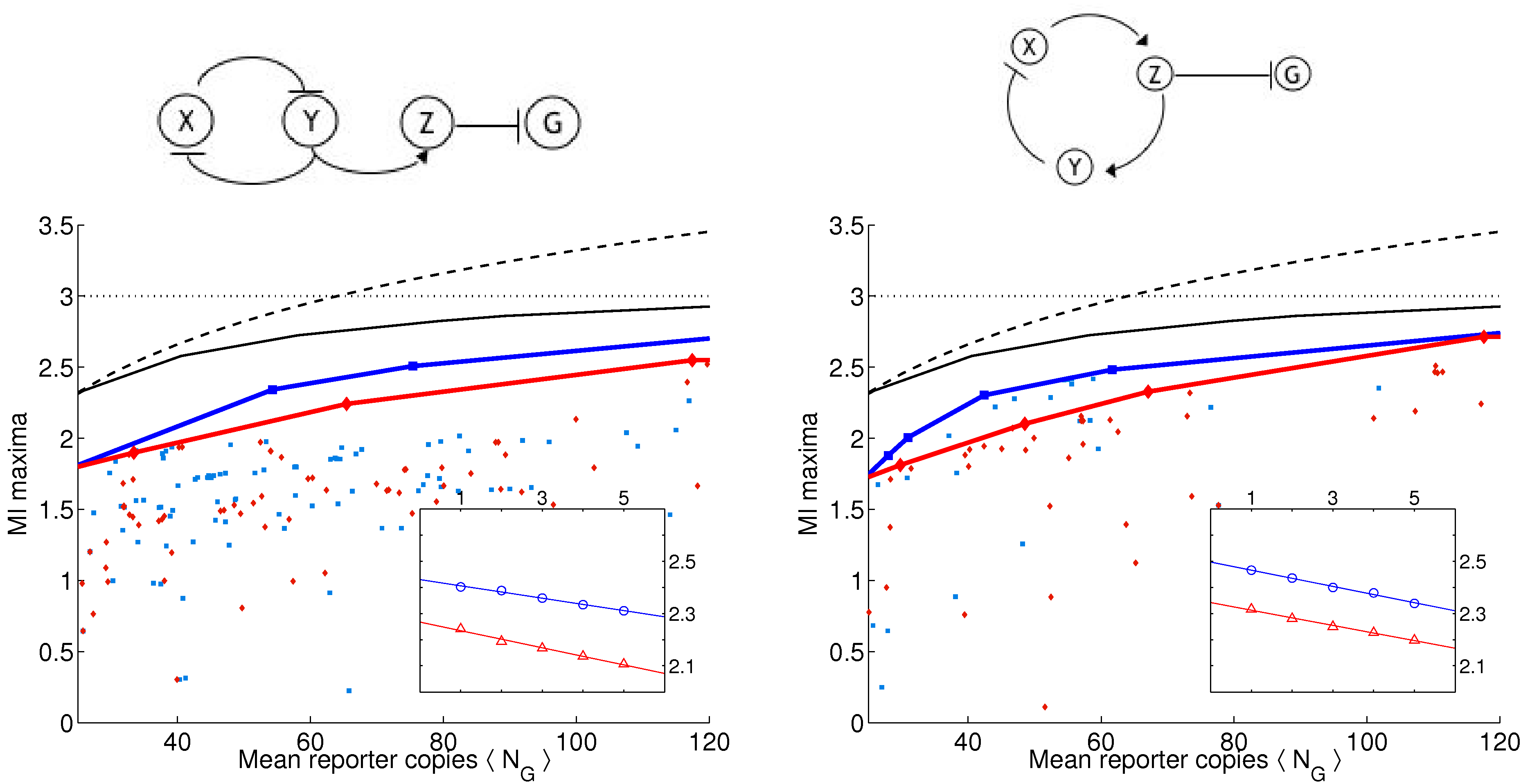

Supplement: Figure S4 — Mutual Information I versus the mean reporter copy number 〈NG〉 for circuits 7 and 8. Insets: Extrapolated 〈I〉 versus the inverse data fraction m as described in the Main Article. (0.45 MB TIF) [file pone.0001077.s005.tif]

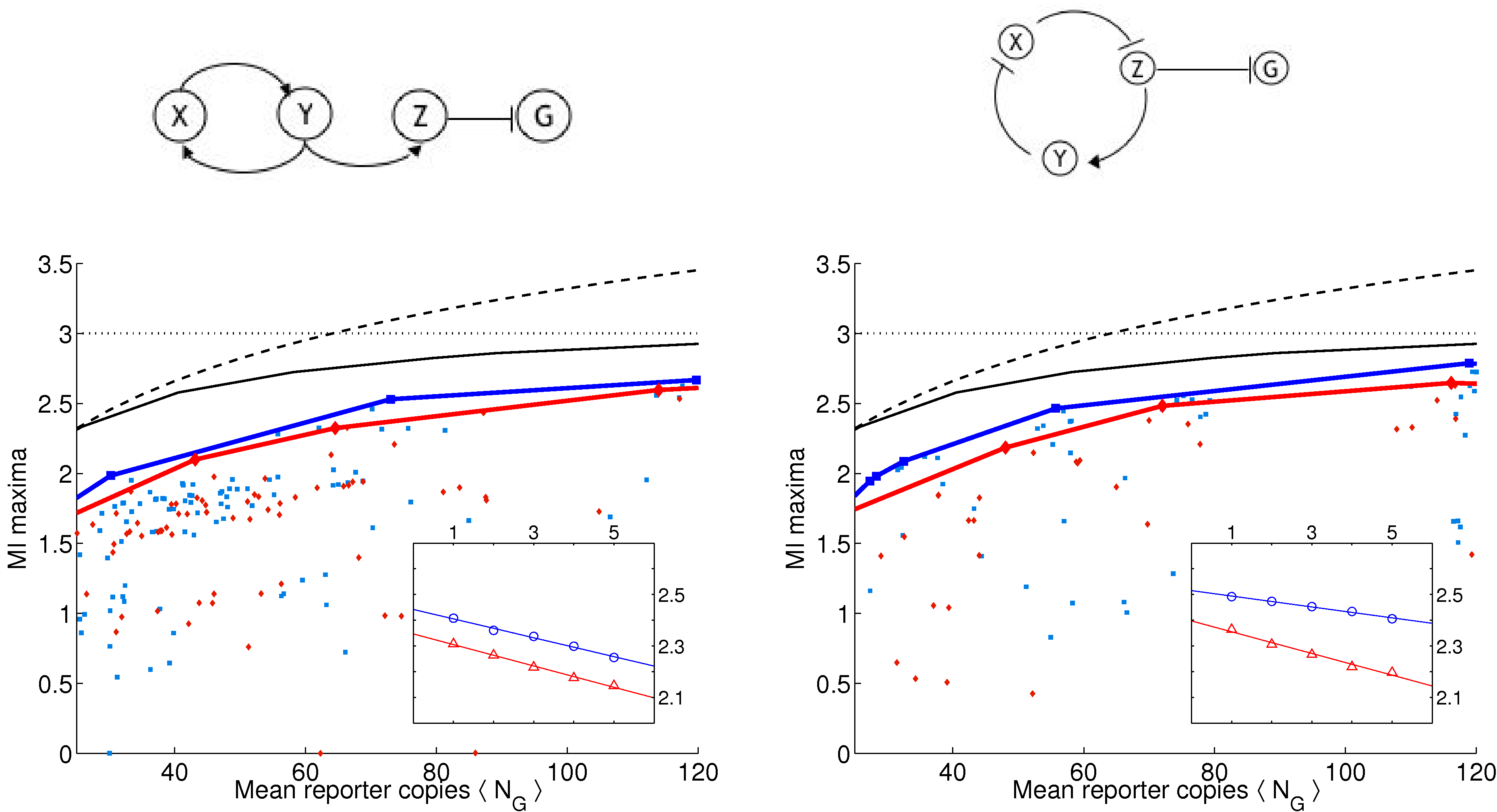

Supplement: Figure S5 — Mutual Information I versus the mean reporter copy number 〈NG〉 for circuits 9 and 10. Insets: Extrapolated 〈I〉 versus the inverse data fraction m as described in the Main Article. (0.46 MB TIF) [file pone.0001077.s006.tif]

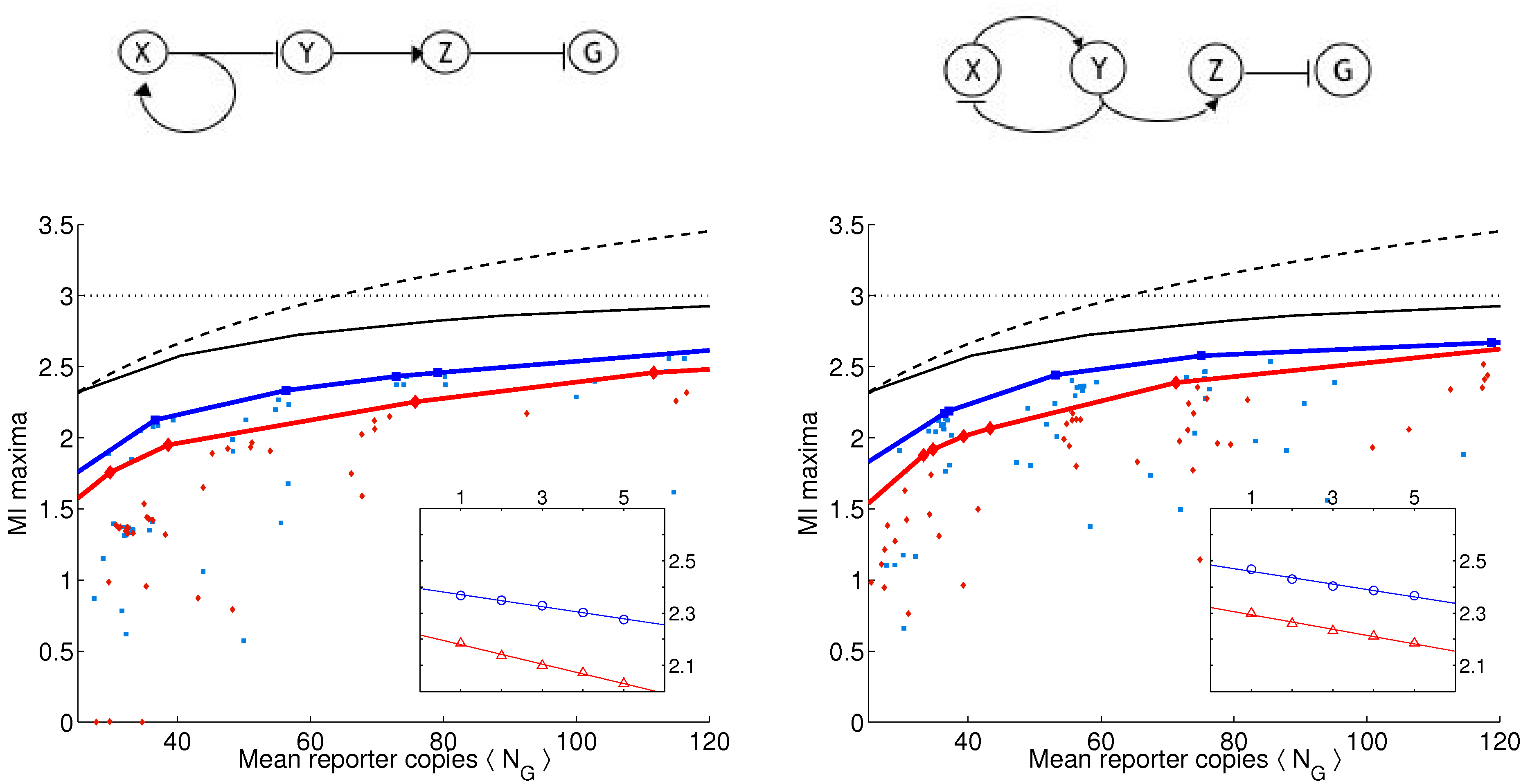

Supplement: Figure S6 — Mutual Information I versus the mean reporter copy number 〈NG〉 for circuits 11 and 12. Insets: Extrapolated 〈I〉 versus the inverse data fraction m as described in the Main Article. (0.45 MB TIF) [file pone.0001077.s007.tif]

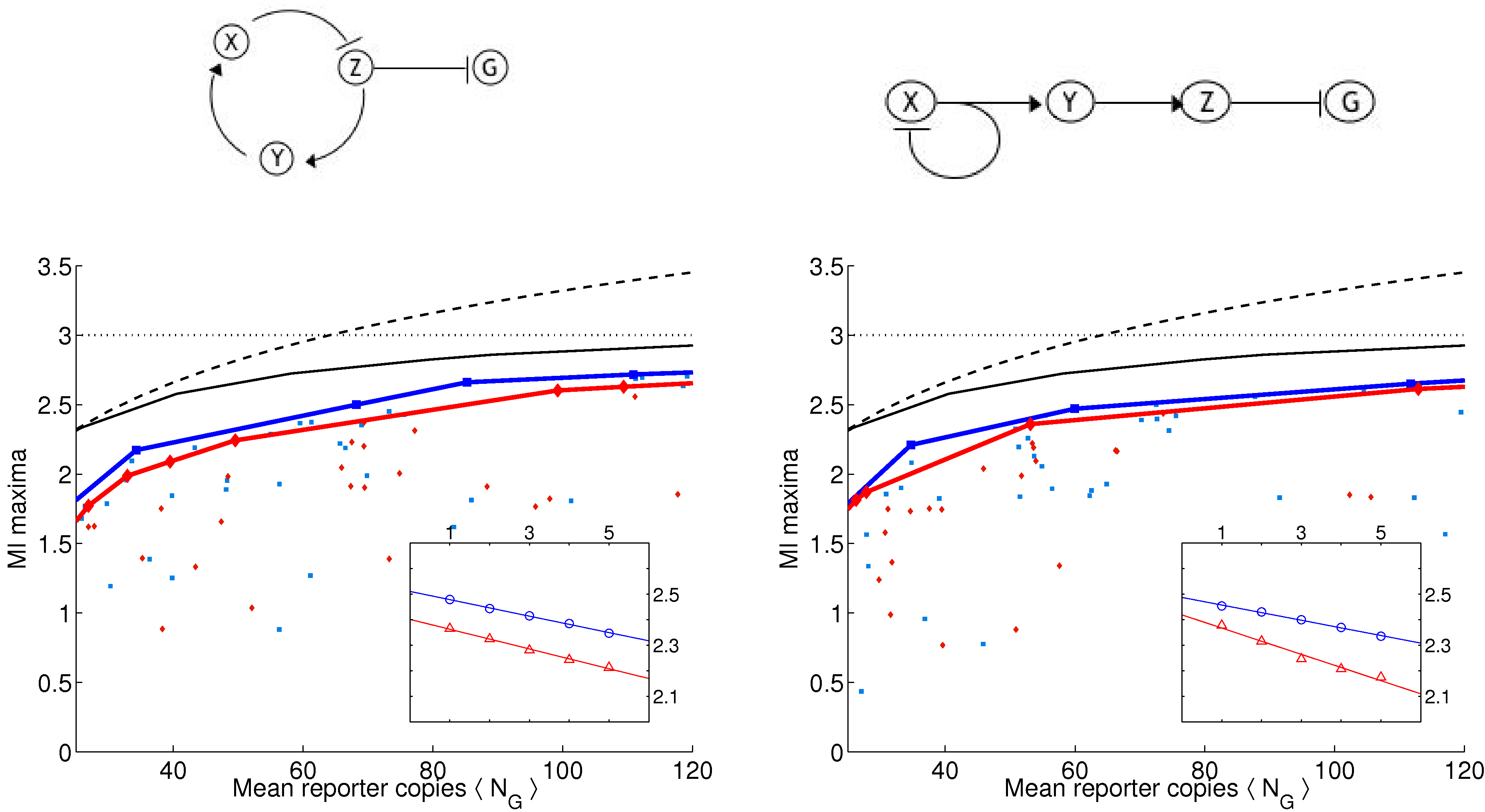

Supplement: Figure S7 — Mutual Information I versus the mean reporter copy number 〈NG〉 for circuits 13 and 14. Insets: Extrapolated 〈I〉 versus the inverse data fraction m as described in the Main Article. (0.44 MB TIF) [file pone.0001077.s008.tif]

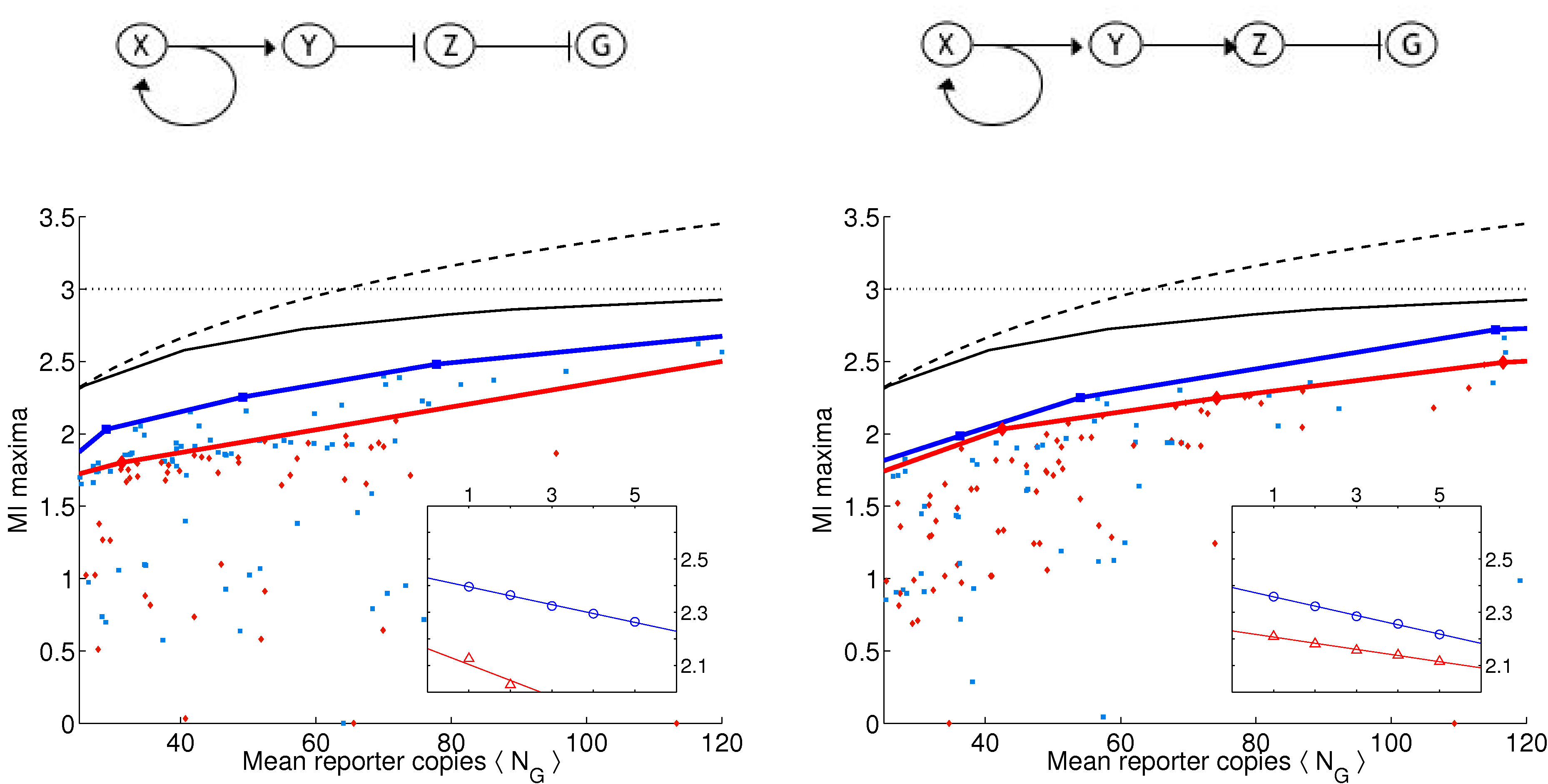

Supplement: Figure S8 — Mutual Information I versus the mean reporter copy number 〈NG〉 for circuits 15 and 16. Insets: Extrapolated 〈I〉 versus the inverse data fraction m as described in the Main Article. (0.43 MB TIF) [file pone.0001077.s009.tif]

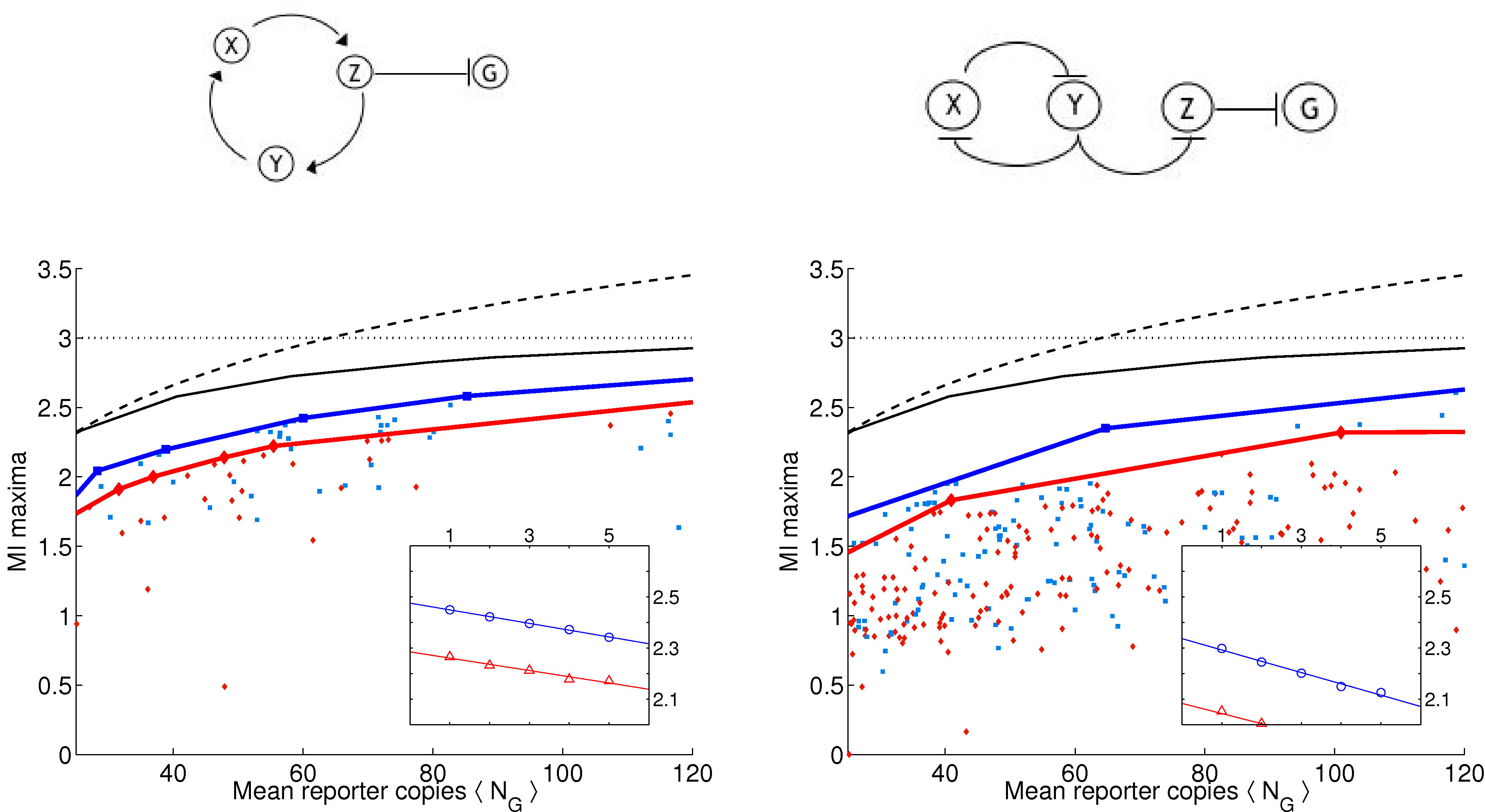

Supplement: Figure S9 — Mutual Information I versus the mean reporter copy number 〈NG〉 for circuits 17 and 18. Insets: Extrapolated 〈I〉 versus the inverse data fraction m as described in the Main Article. (0.47 MB TIF) [file pone.0001077.s010.tif]

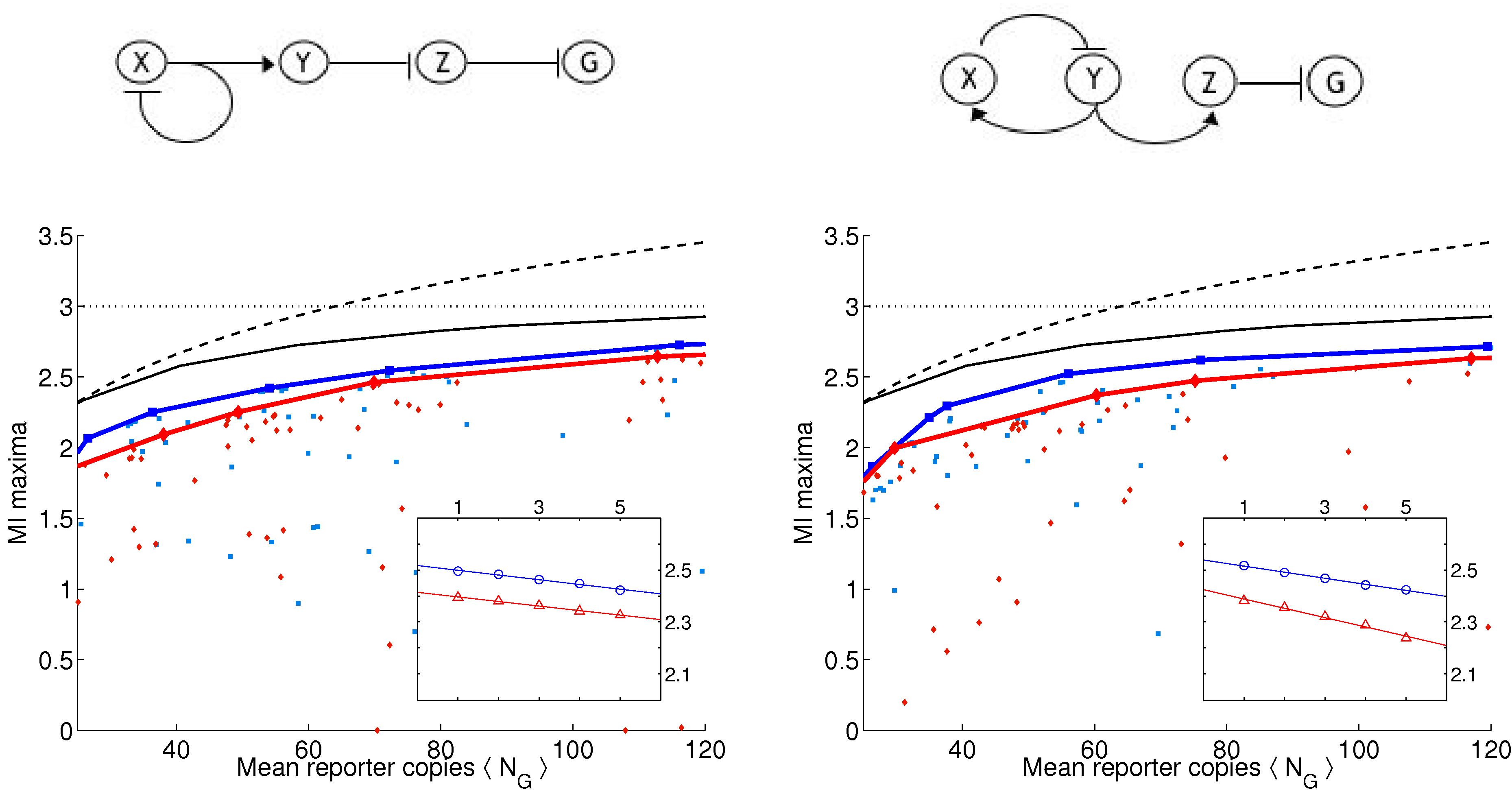

Supplement: Figure S10 — Mutual Information I versus the mean reporter copy number 〈NG〉 for circuits 19 and 20. Insets: Extrapolated 〈I〉 versus the inverse data fraction m as described in the Main Article. (0.45 MB TIF) [file pone.0001077.s011.tif]

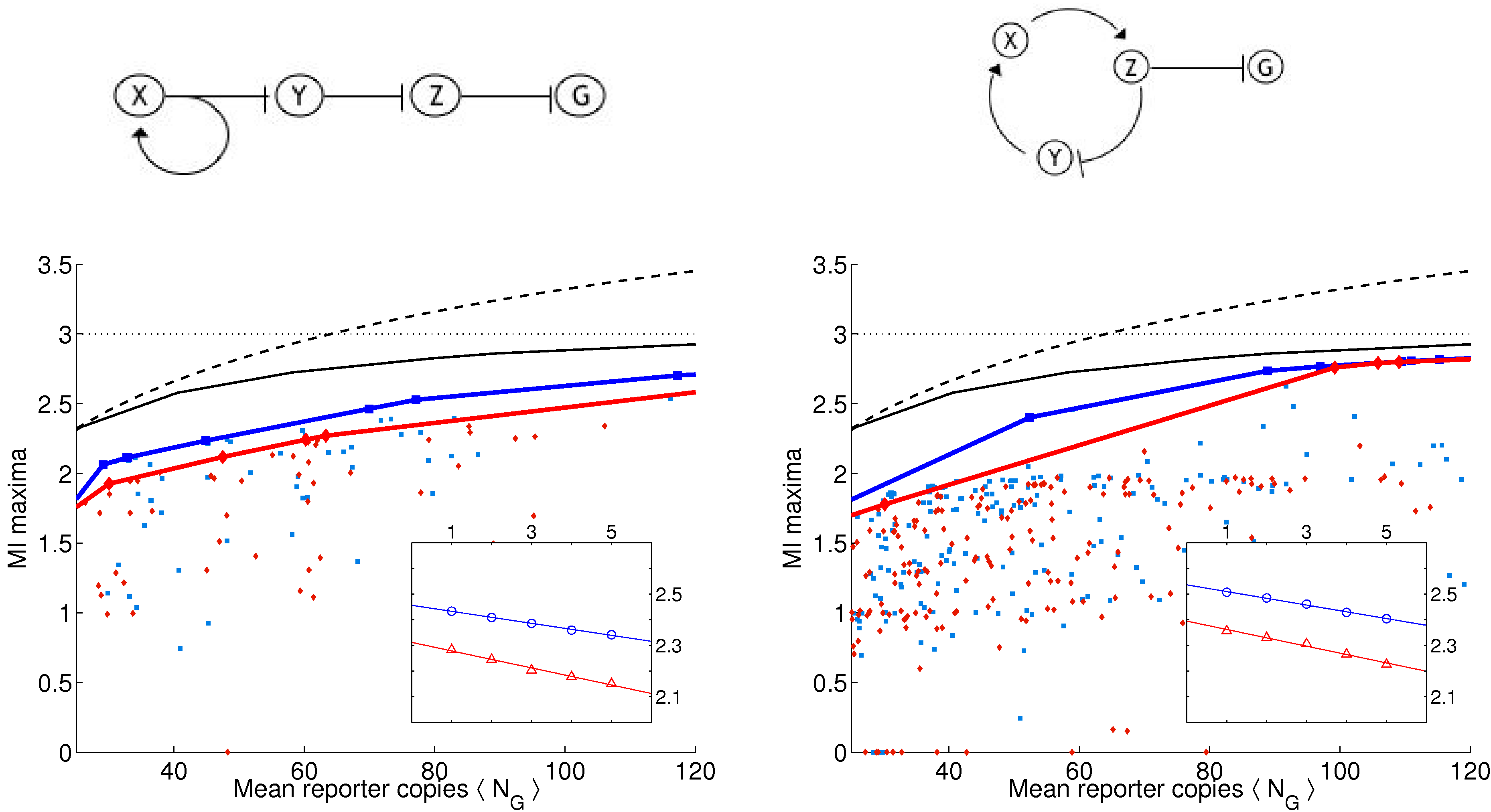

Supplement: Figure S11 — Mutual Information I versus the mean reporter copy number 〈NG〉 for circuits 21 and 22. Insets: Extrapolated 〈I〉 versus the inverse data fraction m as described in the Main Article. (0.46 MB TIF) [file pone.0001077.s012.tif]

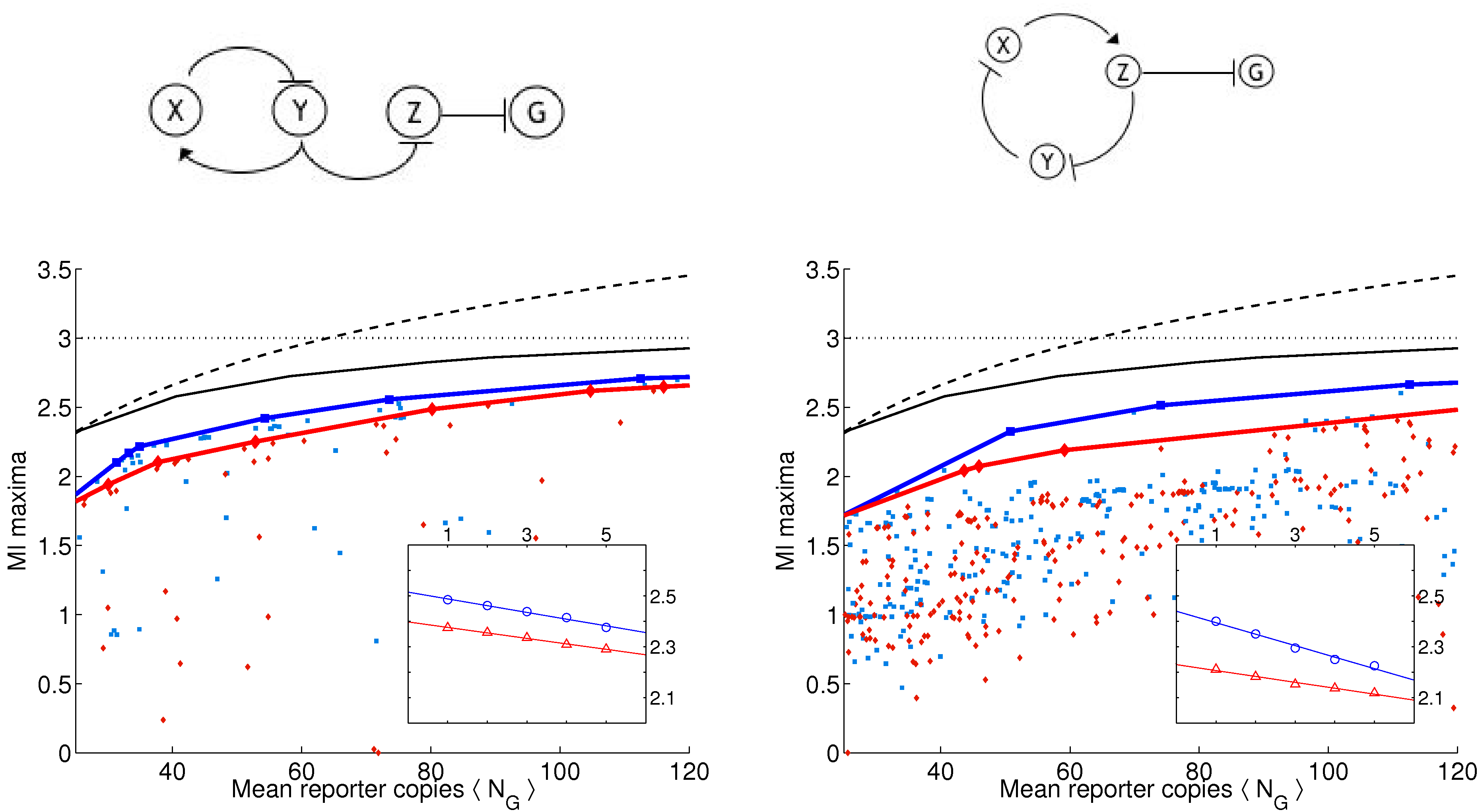

Supplement: Figure S12 — Mutual Information I versus the mean reporter copy number 〈NG〉 for circuits 23 and 24. Insets: Extrapolated 〈I〉 versus the inverse data fraction m as described in the Main Article. (0.49 MB TIF) [file pone.0001077.s013.tif]

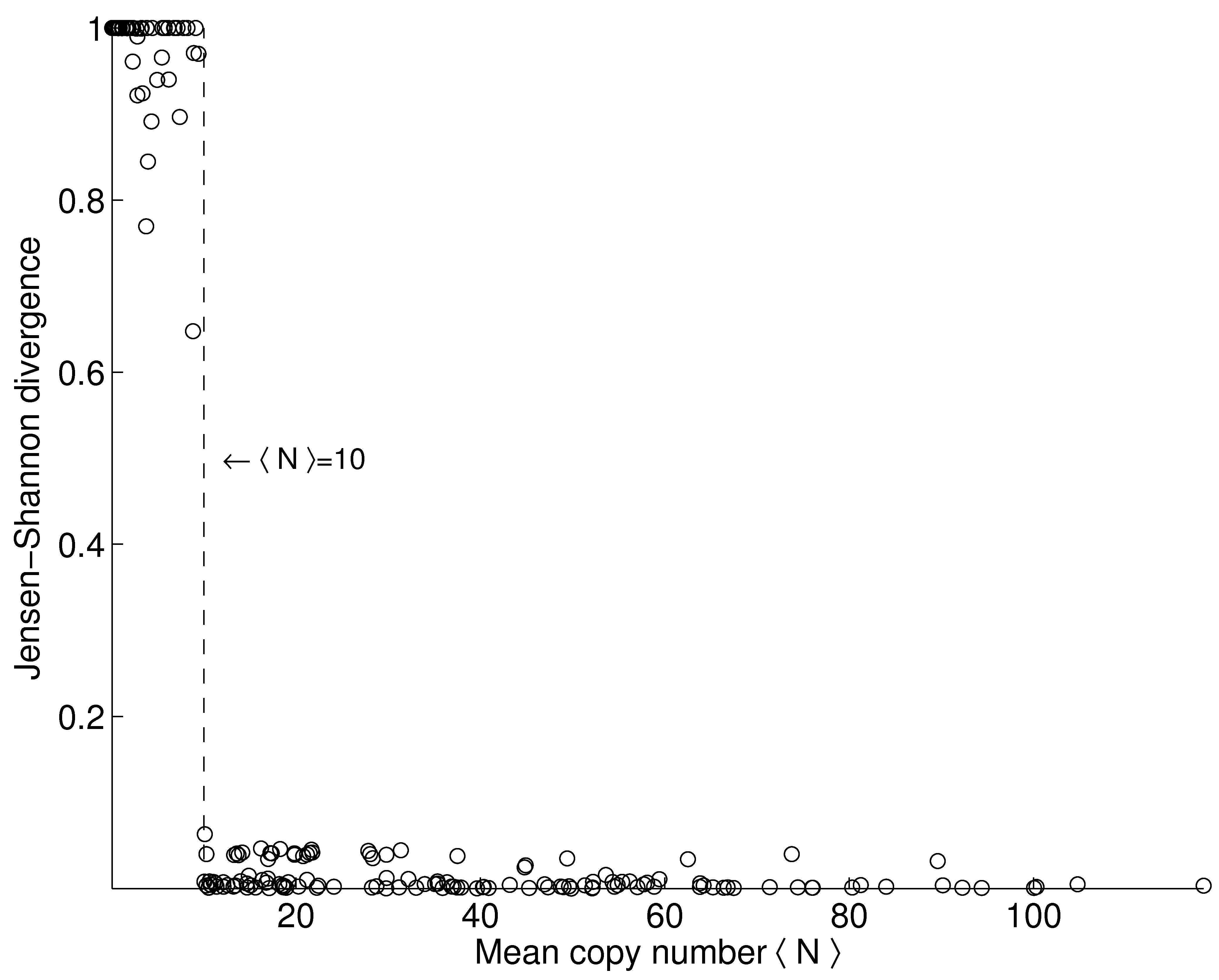

Supplement: Figure S13 — Jensen-Shannon divergence JS Π between distributions obtained by the linear noise approximation and the Gillespie algorithm for multiple circuits and multiple parameterizations plotted as a function of mean copy number. At JS Π = 0, the distributions are identical. There appears to be a sharp threshold at 10 molecules, below which the linear noise approximation does poorly, but above which, the linear noise approximation does well. (0.51 MB TIF) [file pone.0001077.s014.tif]
